# Archaeal Genes Code for GGDEF Domain Proteins With Diguanylate Cyclase Activity

**DOI:** 10.1111/mmi.70117

**Published:** 2026-09-21

**Authors:** Roman Sidorov, Li Li, Ali Dadvar, Irfan Ahmad, Stefan Spring, Jörg Overmann, Ute Römling

**Affiliations:** ^1^ Department of Microbiology, Tumor and Cell Biology, Biomedicum Karolinska Instittuet Stockholm Sweden; ^2^ Leibniz Institute DSMZ‐German Collection of Microorganisms and Cell Cultures Braunschweig Germany; ^3^ Braunschweig University of Technology, Institute for Microbiology Braunschweig Germany

**Keywords:** archaea, cyclic di‐GMP, GGDEF, LUCA, second messenger signaling

## Abstract

Cyclic di‐GMP is ubiquitous in Bacteria, including members of the deepest branching phyla, but has not yet been detected in Archaea. Thus, whether cyclic di‐GMP was present as a signaling nucleotide in the last universal common ancestor (LUCA) of Bacteria and Archaea remains unknown. In this work, bioinformatic analyses and structural modelling identified GGDEF domain proteins in archaeal isolates and encoded by metagenomes of confirmed archaeal origin. In particular, in bacterial model organisms, phenotypic and in vivo assays, in combination with catalytic mutants, suggest that selected archaeal GGDEF domain proteins possess diguanylate cyclase activity. These include the complex REC_S_‐PAS/PAC‐PocR‐GGDEF‐HD‐GYP domain protein of *Methanocella arvoryzae* MRE50, a member of the *Stenosarchaea* order *Methanocellales*. While cyclic di‐GMP signaling proteins are ubiquitous in Bacteria, their presence seems to be more sporadic in Archaea. It is currently unclear whether cyclic di‐GMP signaling proteins have been lost in some lineages, secondarily introduced by horizontal gene transfer into others, or whether both scenarios have occurred.

## Graphical abstract

A biofilm‐like mode of growth is hypothesized to be required for the emergence of cellular life. Occurrence of common biofilm gene products in current Bacteria and Archaea is an indication for their previous presence in the last universal common ancestor LUCA and perhaps even in the first universal common ancestor FUCA. In this work, we demonstrate that GGDEF domain proteins are not only present in Archaea, but are also functional to produce the ubiquitous bacterial second messenger cyclic di‐GMP.

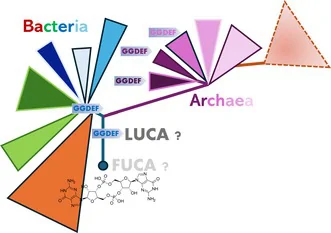

## 1 Introduction

Nucleotide based second messengers are found in all domains of life. For example, cyclic AMP is a long‐established second messenger present in Bacteria, Archaea and Eukaryotes (Botsford and Harman 1992; Leichtling et al. 1986; McDonough and Rodriguez 2011; Rall and Sutherland 1958). The nucleotide (p)ppGpp, found in Bacteria and plants, is synthesized upon inhibition of translation to differentially regulate gene expression (Potrykus and Cashel 2008; Takahashi et al. 2004). Cyclic di‐nucleotides were last discovered in 1987 (Davies et al. 2012; Ross et al. 1987). The cyclic di‐nucleotides regulate ubiquitously fundamental processes such as the sessility‐motility lifestyle switch, osmohomeostasis and phage defense (Corrigan and Grundling 2013; Duncan‐Lowey et al. 2021; Römling et al. 2013; Whiteley et al. 2019). For example, cyclic di‐AMP regulates osmohomeostasis throughout Bacteria and Archaea (Corrigan et al. 2013). Thereby, the DAC diadenylate cyclase domain, a short domain of 123 amino acids (Witte et al. 2008) has diversified substantially during evolution and is linked to a broad range of different sensory domains sensing unrelated signals (Corrigan and Grundling 2013; Römling 2008). Many nucleotide second messengers have dedicated synthases, but others (such as 2′,3′ cyclic NMPs and Ap_3_A/Ap_4_A) are produced as non‐canonical byproducts of enzymatic reactions (Fontaine et al. 2018; Zamecnik et al. 1966). Besides enzymatic synthesis, there is emerging evidence that nucleotide‐based signaling molecules can also be synthesized non‐enzymatically in mineral‐catalyzed chemical reactions consistent with an ancient and possibly even prebiotic role in the emergence of life (Römling 2023b; Wu et al. 2025).

Here, we have focused on the phylogenetic distribution of cyclic di‐guanylate (cyclic di‐GMP). In Bacteria, cyclic di‐GMP frequently regulates the transition between the fundamental life styles sessility (biofilm formation) and motility including associated physiological traits such as antimicrobial and stress tolerance (Dayton et al. 2020; Simm et al. 2004). Thereby, single cell level regulation by cyclic di‐GMP leads to a substantial bi‐ and (most likely) multi‐modality of the bacterial population where individual cells are differentially prepared to respond to environmental challenges and altered growth conditions (Grantcharova et al. 2010; Kulasekara et al. 2013). In the ecological context, the impact of cyclic di‐GMP spans from intra‐strain to complex intrakingdom and interkingdom interactions. Cyclic di‐GMP signaling modulates the association of microbes with abiotic and biotic surfaces, cell–cell interactions and affects development of pro‐ and eukaryotic organisms and cells including the development of immune cells (Hu et al. 2024; Peng et al. 2020; Petersen et al. 2019; Römling 2023a; Ruiz et al. 2012). Although cyclic di‐GMP can regulate the cell cycle, dysregulation of cyclic di‐GMP signaling has rarely been found to be lethal or to impair growth under laboratory conditions (Christen et al. 2006; Liu et al. 2020; Lori et al. 2015). The relevance of cyclic di‐GMP signaling for Bacteria is reflected by the nearly ubiquitous occurrence of enzymatic domains in all bacterial phyla to direct the turnover of cyclic di‐GMP. Thereby, the GGDEF domain mediates diguanylate cyclase activity (Schirmer and Jenal 2009). The EAL and the HD‐GYP domain possesses cyclic di‐GMP specific phosphodiesterase activity with pGpG and GMP as major product outputs, respectively (Galperin et al. 2001; Ryan et al. 2007; Schmidt et al. 2005; Simm et al. 2004; Tamayo et al. 2005). Besides their broad phylogenetic distribution, cyclic di‐GMP turnover domains are abundantly encoded by bacterial genomes (Römling et al. 2013, 2005). This multitude of turnover domains which are linked to a diversity of (mostly N‐terminally located) signaling domains highlights the need for complex integration of a multitude of diverse signals on the post‐translational level. This diversity of signaling modules adds an additional regulatory level beyond transcriptional and post‐transcriptional regulatory mechanisms. Local signaling via protein–protein interactions versus global signaling by highly active diguanylate cyclases further adds to the mechanistic diversity of signal transduction by this diffusible second messenger.

GGDEF, EAL, and HD‐GYP domains, superfamilies in their own rights with consistent enzymatic activity, show substantial diversifying radiation within one genome. The amino acid diversity between two domain members can thereby nearly equal the diversity between randomly selected members from the bacterial kingdom (Galperin and Chou 2022; Römling et al. 2023; Schirmer and Jenal 2009). With N‐terminal signal transduction domains and the scaffold function of the catalytic domains being mostly determinative for the sequence diversity, the substrate and product specificities of the domains are surprisingly conserved (Schirmer 2016). Only a subset of GGDEF domains with distinct scaffolds synthesize predominantly the alternative signaling molecule cyclic GMP‐AMP (Hallberg et al. 2019; Nelson et al. 2015). In parallel, certain HD‐GYP domains can hydrolize cyclic GMP‐AMP (Gao et al. 2015). Further more, product, substrate, and non‐binding variants without catalytic activity evolved in the three domain superfamilies.

Biofilms are today the predominant life style of Bacteria and Archaea (Flemming and Wuertz 2019). Matrix‐protected microbial cells closely associated with abiotic surfaces, primordial biofilms, have been hypothesized to be a prerequisite for the origin of life (Martin and Russell 2003; Römling 2023b; Trevors 2011). Exclusively sessile cells, however, are only partially able to survive and spread. Consequently, regulation of biofilm formation might have arisen in parallel in the last universal common ancestor (LUCA) of Bacteria and Archaea or even earlier. Based on bioinformatic analyses, we have previously predicted that cyclic di‐GMP signaling and its turnover proteins are present in deeply branching Bacteria and Archaea (Römling 2023b). In order to gain evidence for the presence of cyclic di‐GMP signaling in LUCA, we were therefore wondering whether cyclic di‐GMP signaling is found in Archaea (and regulating the motility/sessility lifestyle switch). A valid argument for primordial occurrence of the cyclic di‐GMP signaling molecule is the presence of the catalytic domains in the two prokaryotic domains of life. We were therefore investigating whether cyclic di‐GMP signaling can occur in Archaea. To this end, bioinformatic analyses and experimental validation showed that diverse GGDEF domains with diguanylate cyclase activity occur in Archaea with their genes expressed in archaeal isolates.

## 2 Results

### 2.1 Bioinformatic Identification of GGDEF Domains in Archaeal Isolates

BLAST search with the GGDEF domain containing diguanylate cyclase AdrA of *S. typhimurium* ATCC 14028 as query yielded an extensive list of potential GGDEF domain proteins encoded by Archaea. BLAST searches with AdrA as a query have previously been proven to robustly recognize GGDEF domains (Liu et al. 2020). The vast majority of identified GGDEF domains was, however, found encoded by metagenome associated genomes (MAGs) contigs with a genuine archaeal origin challenging to proof. Therefore the list was screened for GGDEF proteins encoded by genomes of archaeal isolates that are maintained as a pure culture. Indeed, we identified the GGDEF proteins WP_012035199.1 (GGDEF_MRE50), WP_339106355.1 (GGDEF_KLK7), and WP_363463662.1 (GGDEF_E3) as encoded by archaeal genomes of *Methanocella arvoryzae* MRE50 (Sakai et al. 2010), *Haloterrigena salinisoli* KLK7 (Dong et al. 2024), and *Halogeometricum borinquense* E3 (Nagar et al. 2024). The three GGDEF domain proteins are encoded by isolates that are members of the Stenosarchaea, with *M. arvoryzae* MRE50 belonging to the order *Methanocellales*, while the extreme halophilic *H. salinisoli* KLK7 and *H. borinquense* E3 belong to the Halobacteria (Figure 1A). In detail, *H. salinisoli* KLK7 and *H. borinquense* E3 are categorized to the order *Natrialbales* (family *Natrialbaceae*) and *Haloferacales* (family *Haloferacaceae*), respectively (Figure S1). In conclusion, GGDEF domain proteins were identified in only few of the fully sequenced genomes which can indicate that GGDEF domains are far less abundant within archaeal compared with bacterial genomes.

**FIGURE 1:**
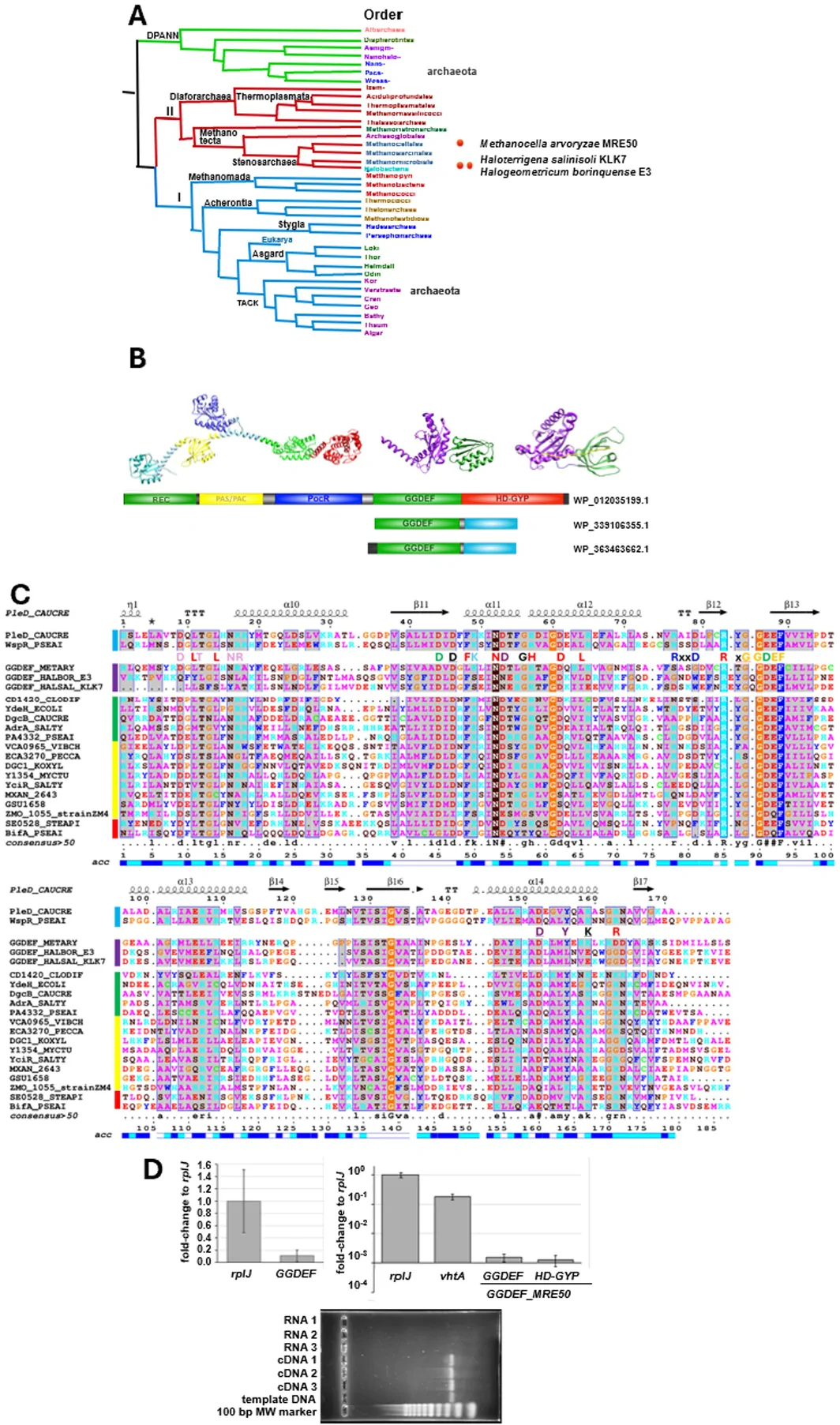
Initial characterisation of GGDEF domain protein from archaeal isolates *M. arvoryzae* MRE50, *H. salinisoli* KLK7 and *H. borinquense* E3. (A) Phylogenetic tree of Archaea with phylogenetic position of isolates encoding GGDEF domain proteins. Phylogenetic tree has been redrawn from (Aouad et al. 2022). (B) Structural models and domain structure of GGDEF domain proteins GGDEF_MRE50 (WP_012035199.1) from *M. arvoryzae* MRE50, GGDEF_KLK7 (WP_339106355.1) from *H. salinisoli* KLK7 and GGDEF_E3 (WP_363463662.1) from *H. borinquense* E3. Structural models have been calculated with AlphaFold 3 (Jumper et al. 2021) with colors according to domain structure. (C) Alignment of GGDEF domains of proteins GGDEF_MRE50 from *M. arvoryzae* MRE50, GGDEF_KLK7 from *H. salinisoli* KLK7 and GGDEF_E3 from *H. borinquense* E3 with GGDEF domains from well investigated catalytically active GGDEF domains; PleD from *Caulobacter vibrioides* CB15 (CC_2462, PleD_CAUCRE), WspR from *Pseudomonas aeruginosa* PAO1 (PA3702, WspR_PSEAI) and class I, II, and III GGDEF domains. GGDEF domain starts with aspartate at arbitrary position 9. Conserved signature motifs are indicated: light plum, wide turn; red, substrate interacting residues; green, Mg^2+^ binding; light blue, stabilizing the transition state; blue, allosteric I‐site; violet, probable dimer interface; yellow, GG[D/E]EF motif; underlined, salt bridge. Light blue side bar, reference domains; green, catalytically active GGDEF domains; yellow, domains with unconventional signature motifs; red, catalytically inactive GGDEF domains. Protein designation in Supporting Information and methods. Alignment was created with ClustalX 2.1 (Higgins and Sharp 1988), manually curated in GeneDoc (Nicholas et al. 1997) and visualized with ESPript 3.0 (Robert and Gouet 2014). Secondary structure of PleD from 2V0N (RCSB Protein Data Bank, RCSB PDB). *acc*, accessibility. (D) Quantitative RT‐PCR of the expression level of the GGDEF genes of *M. arvoryzae* MRE50 and *H. salinisoli* KLK7. Normalisation was to the expression level of the ribosomal protein L10 gene *rplJ* which showed an average C_t_ value of 12.5 and 23.6 respectively. The average C_t_ values of the GGDEF genes were 22 and 26.1, respectively. Lower row, PCR amplification of the 251 bp internal fragment of *rplJ* for quality control of total RNA preparation. cDNA and DNA template served as positive controls. GeneRuler^TM^ 100 bp molecular weight (MW) marker from Fermentas.

### 2.2 Chromosomal Context of GGDEF Domain Proteins

We subsequently assessed whether proteins highly similar to GGDEF_MRE50, GGDEF_KLK7 and GGDEF_E3 can be found encoded by MAGs representing completely sequenced genomes or contigs. Indeed, the genome sequences of two other *Methanocella* sp., *Methanocella* sp. MCL‐LM and *Methanocella* sp. SMAG_U12379 code for WP_424359454.1 and HMK46096.1. These proteins are homologous to GGDEF_MRE50 over the entire length with overall amino acid identity/similarity of 82.5/87.0% and 52.2/68.7%, respectively. The high identity of the GGDEF proteins in combination with the conservation of the domain structure points to vertical transfer from a common ancestor. While WP_424359454.1 is encoded in a similar genetic context as GGDEF_MRE50, only the immediate gene environment is preserved for HMK36096.1 (Figure S2A). Isolate *M. avoryzae* MRE50 has been recovered from the soil of an Italian rice field, *Methanocella* sp. MCL‐LM from the soil of a sandy desert in Israel and *Methanocella* sp. SMAG_U12379 from wetland soil in Colombia. Thus highly homologous GGDEF domain proteins encoded by genomes of *Methanocella* spp. were derived from distinct geographical regions. This indicates that acquisition/maintenance of a chromosomally encoded GGDEF_MRE50 homolog has not been an isolated or accidental event, but GGDEF proteins are more commonly present in the *Methanocella* genus. In Bacteria, abundance of cyclic di‐GMP turnover proteins in one genome is positively correlated with genome size. The genome size of the three *Methanocella* isolates spans from 3.18 to 3.61 Mbps which is in the upper size range of archaeal genomes. The 5.75 Mb genome of *Methanosarcina acetivorans* is currently recognized as the largest archaeal genome (Kellner et al. 2018), however, it does not encode a GGDEF domain protein as indicated by BLAST search (data not shown).

The GGDEF domain proteins GGDEF_KLK7 of *H. salinisoli* KLK7 and GGDEF_E3 of *H. borinquense* E3 have an amino acid identity/similarity of 69.6/79.3%. A homologous, but truncated protein of 174 amino acids is found in a halite metagenome (Finstad et al. 2017). Again, the isolates derived from geographically distant areas. While *H. salinisoli* KLK7 has been isolated from a salt lake in the Qinghai Province, China, *H. borinquense* E3 has been recovered from solar salterns, a hypersaline sediment at Marakkaman, Tamil Nadu, India. Genome size of the two strains spans from 3.8 (excluding four plasmids) to 4.0 Mbps, respectively, sizes at the upper end of archaeal genomes. Despite the high identity/similarity, the genomic context of the two GGDEF domain protein genes is different (Figure S2B). The GGDEF proteins are encoded on the 212 kbp plasmid “unknown 3” of *H. salinisoli* KLK7 and on a 240 kbp chromosomal contig of *H. borinquense* E3. In conclusion, the occurrence of GGDEF domain protein genes in (closely) related archaeal genomes recovered at significant geographical distance indicates vertical transmission, while their occurrence on plasmids indicates the potential for horizontal transmission in similar ecological niches.

### 2.3 Archaeal GGDEF Proteins Possess a Diverse Domain Structure

Sequence and structural analyses of the domain structure showed that the 810 amino acid long GGDEF_MRE50 is a multidomain protein with at least a REC, PAS/PAC, PocR, GGDEF, and HD‐GYP domain (Figure 1B). REC receiver domains are conventionally the N‐terminal domains of response regulators as part of bacterial two component systems (Foulkes et al. 2025; Wadach et al. 2025). REC receiver domains can be phosphorylated at a conserved aspartate at the position equivalent 56 in order to allosterically modulate the activity of a downstream effector domain that include GGDEF, EAL. and HD‐GYP domains (Paul et al. 2004; Rao et al. 2008; Wigren et al. 2014). The REC domain of GGDEF_MRE50 is structurally similar to conventional phosphotransfer receiver domains. Interestingly, AlphaFold 3 modelling and structural comparison to experimentally determined REC domains indicated that the amino acid sequence does not consecutively align (Figure S3). Nevertheless, two aspartates, D14 and D58, are in a position to serve as receivers of a phosphoryl group. However, the D/E_−44_D_−43_DK_+50_ spatial motif context present in canonical receiver domains is absent. Thus the N‐terminal REC domain was designated REC_S_ to reflect the predominantly structural homology. Diverse classes of PAS (Per‐Ant‐Sim) domains, short signaling domains that can sense a wide variety of chemical and physical signals (Dayton et al. 2020), are frequently linked to GGDEF, EAL and HD‐GYP domains (Galperin and Chou 2022; Schirmer 2016). PocR is a PAS‐like‐fold ligand sensing domain that has been shown to bind hydrocarbons such as 1,2 propanediol (Anantharaman and Aravind 2005; Teng et al. 2024). The REC_S_, PAS/PAC and PocR signaling domains and the GGDEF domain are consecutively connected by α‐helix‐like linkers. In particular, the PocR domain is connected with the GGDEF domain by a 57 amino acid long linker that forms a coiled‐coil structure upon dimerization. Coiled‐coil linkers can flexibly modulate their dimerization upon signal perception and are thereby determinative for the regulation of downstream domain activities that require dimerization and correct positioning of domains. As the adenylate cyclase domain, the structurally homologous GGDEF domain becomes catalytically active upon dimerization (De et al. 2009; Gourinchas et al. 2017; Schirmer 2016).

On the other hand, the 262 amino acid long GGDEF_KLK7 (alternative longer open reading frame used) and 272 amino acid long GGDEF_E3 (alternative longer open reading frame used) possess an unconventional GGDEF‐(domain of unknown function) structure with a signaling/sensing/interacting domain at the C‐terminal end of the protein.

In conclusion, archaeal GGDEF domain proteins are linked to N‐ and C‐terminal established and novel signaling domains.

### 2.4 Conserved Signature Motifs in Archaeal GGDEF Domains

In order to predict by bioinformatic means whether the GGDEF domains possess diguanylate cyclase activity, the GGDEF domains of GGDEF_MRE50, GGDEF_KLK7, and GGDEF_E3 were aligned with previously characterized reference and class I, II, and III GGDEF domains (Figure 1C; (De et al. 2009; Liu et al. 2020; Paul et al. 2004; Römling et al. 2013)). GGDEF domains start with a wide turn containing the DxLT motif (Schirmer 2016). Class I GGDEF domains, including the GGDEF domains of the reference diguanylate cyclases PleD and WspR, are unambiguously predicted to be dinucleotide cyclases due to full conservation of signature amino acids, including residues involved in catalysis. Importantly, those GGDEF domains have been experimentally characterized as diguanylate cyclases. The bioinformatic assignment of evolved class II GGDEF domains is ambiguous due to distinct alteration in key signature amino acids, resulting in noncanonical motifs, but their domains have been experimentally verified to be dinucleotide, and predominantly diguanylate, cyclases. GGDEF domains with the GGDEF motif altered to A/SGDEF belong to this class (Fineran et al. 2007). This class also contains the cyclic GMP‐AMP synthesizing GGDEF domains, which are characterized by a signature serine replacing aspartate at the otherwise conserved xxGD motif (Hallberg et al. 2019). A significant number of signature amino acids are not conservatively replaced in class III GGDEF domains, and/or catalytic activity has not been experimentally demonstrated despite canonical motifs (Holland et al. 2008; Kuchma et al. 2007). While this classification does not allow unambiguous prediction of catalytic activity, it provides a first rational assessment. Detailed comparative assessment of the GGDEF domains of GGDEF‐MRE50, GGDEF_KLK7, and GGDEF_E3 showed that the GGDEF domain of GGDEF_MRE50 possesses the GGDEF motif and other signature amino acids essential for catalytic activity, indicating that this GGDEF domain is potentially catalytically active (Figure 1C). On the other hand, the GGDEF domains of GGDEF_KLK7 and GGDEF_E3 possess a semi‐conserved GGDEF motif, GQGDEF, and also several other signature amino acids in the domain are not conserved.

In conclusion, as two of the three genomes of archaeal isolates code for GGDEF domains with non‐canonical motifs, GGDEF domains in Archaea might have undergone significant domain evolution. Domain evolution is not uncommon as bacterial GGDEF domains can possess solely GTP or cyclic di‐GMP binding activity or do not bind the substrate and product at all (Christen et al. 2005; Lee et al. 2007; Römling et al. 2023; Suzuki et al. 2006). GGDEF domain evolution might have gone in a different direction and/or is accelerated in Archaea.

### 2.5 GGDEF Domain Protein Genes Are Expressed in Archaeal Isolates

As the presence of GGDEF domain proteins encoded by archaeal isolates was confirmed, we were wondering whether the genes are expressed. To this end the expression of GGDEF protein genes was assessed by qRT‐PCR. The essential ribosomal protein L10 gene *rplJ* was selected as the reference gene and positive control for both isolates, *M. arvoryzae* MRE50 and *H. solinisoli* KLK7. The GGDEF_MRE50 is a complex protein with at least five different domains which might allow for the presence of internal promoters and differential processing of the mRNA transcript of the open reading frame. Therefore the GGDEF and HD‐GYP domain encoding nucleotide sequences were targeted for assessment. The steady state levels of the *GGDEF_MRE50* mRNA, as assessed based the GGDEF and HD‐GYP mRNA sequences, was 0.15% of the level of *rplJ* and 0.77% of the level of *vhtA* encoding the large subunit of the membrane‐bound F420‐nonreducing hydrogenase critical for methanogenesis (Figure 1D). *VhtA* has been selected to assess the expression of a potentially essential gene with less abundant copy number compared to ribosomal protein genes. In *H. salinisoli* KLK7, the *GGDEF_KLK7* mRNA steady state level was 10.7% of the *rplJ* level. Thus in the two isolates the GGDEF gene is expressed to contribute to archaeal physiology and metabolism.

### 2.6 Alphafold 3 Models of Archaeal GGDEF Domain Proteins Are Coherent With Experimentally Verified GGDEF Domain Proteins

Closest 3D homologs of the AlphaFold 3 structural models were retrieved using DALI and FoldSeek. The model structures of the three GGDEF domains from GGDEF_MRE50, GGDEF_KLK7, and GGDEF_E3 proteins showed highest structural homology to experimentally determined crystal structures of catalytically active GGDEF domains: to GcbC from *Pseudomonas fluorescens* (PDB: 5EUH), DgcB from *Caulobacter crescentus* (PDB: 6TTS), and YfiN from *Pseudomonas aeruginosa* (PDB: 4IOB), respectively (Figure 2A). Overlap of the structural models with the experimentally determined structures verified the significant similarity in the secondary structures with unstructured loops deviating most (Figure 2A). The β‐hairpin containing the catalytic site motif GGDEF was coherent between the GGDEF_MRE50 structural model and the 5EUH GGDEF crystal structure (Figure 2B). On the other hand, the β‐hairpin deviated significantly for GGDEF_KLK7/6TTS and GGDEF_E3/4IOB as already indicated by the GQGDEF motif compared to the conventional GGDEF motif.

**FIGURE 2:**
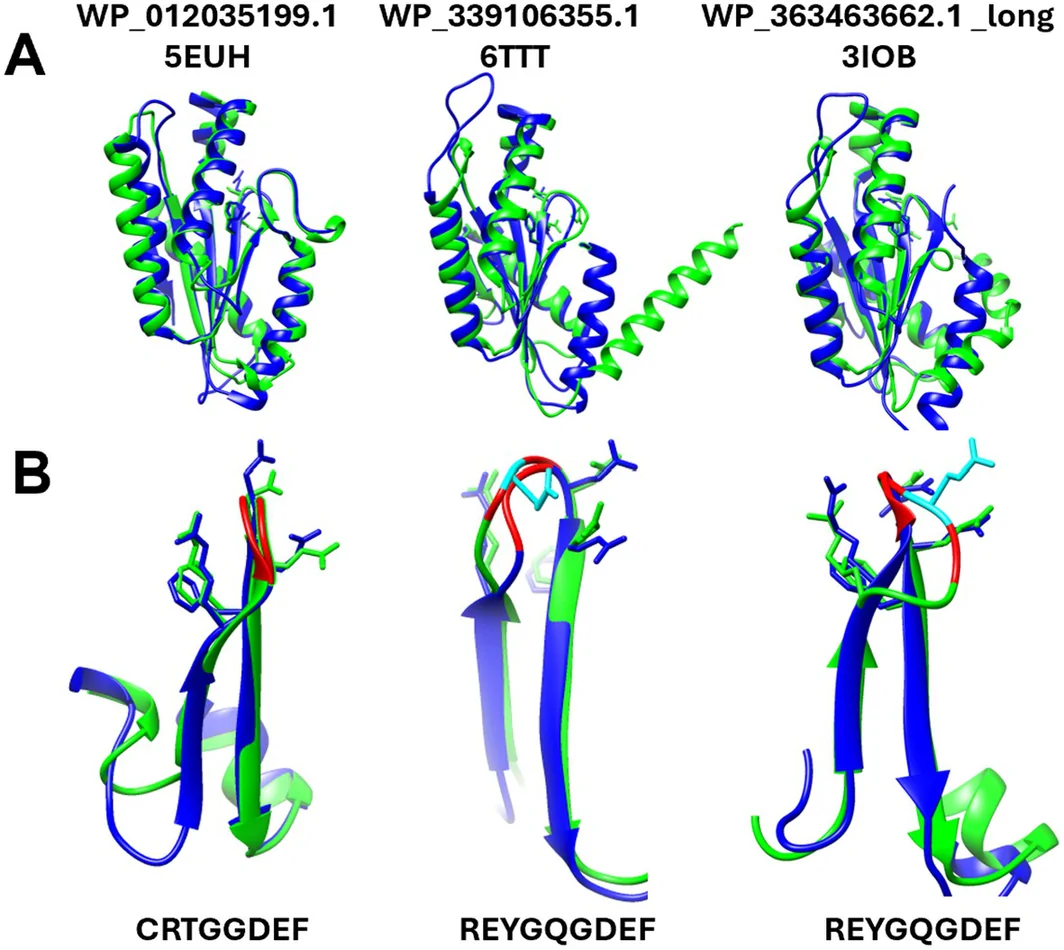
Structural models of GGDEF domains of proteins GGDEF_MRE50 (WP_012035199.1) from *M. arvoryzae* MRE50, GGDEF_KLK7 (WP_339106355.1) from *H. salinisoli* KLK7 and GGDEF_E3 (WP_363463662.1) from *H. borinquense* E3 overlayed with most homologous experimentally determined crystal structures of catalytically active GGDEF domains. (A) Structural models (in green) of GGDEF domains of proteins GGDEF_MRE50 from *M. arvoryzae* MRE50, GGDEF_KLK7 from *H. salinisoli* KLK7 and GGDEF_E3 from *H. borinquense* E3 were determined by AlphaFold 3 (Jumper et al. 2021). Overlay with 5EUH (GcbC of *Pseudomonas fluorescens* ) (Dahlstrom et al. 2015), 6TTS (DgcB from *C. vibrioides* CB15) and 4IOB (PA1120/YfiN/TpbB from *Pseudomonas aeruginosa* PAO1 (Giardina et al. 2013)) in blue, respectively. Structural homologs to the structural models were searched for with DALI (Holm et al. 2023) and FoldSeek (van Kempen et al. 2024) and subsequently overlayed with MatchMaker in Chimera 1.18 (Pettersen et al. 2004). (B) Close‐up on the overlay between the structural models (green) of GGDEF domains of proteins GGDEF_MRE50 from *M. arvoryzae* MRE50, GGDEF_KLK7 from *H. salinisoli* KLK7 and GGDEF_E3 from *H. borinquense* E3 and experimentally determined structures (blue) 5EUH, 6TTS, and 4IOB, respectively focusing on the β‐hairpin with the GGDEF motif. Amino acids of the GGDEF motifs are displayed with their side chains with glycine in red and inserted glutamine Q in light blue.

### 2.7 Archaeal GGDEF Domain Proteins Possess Homologs in Bacteria

After separation into different lineages beyond LUCA, members of the Archaea and Bacteria domains of life have continuously exchanged genetic material throughout their evolution. Indeed, novel archaeal orders had arisen through extensive horizontal gene transfer from Bacteria (Gophna and Altman‐Price 2022). We were therefore wondering about the closest relatives of the GGDEF domains from the archaeal isolates. As reported above, proteins with the highest similarity to the *M. arvoryzae* MRE50 REC_S_‐PAS/PAC‐PocR‐GGDEF‐HD‐GYP domain protein over the entire length are found in other *Methanocella* spp. (Figure S2A). We were subsequently assessing whether closely related homologs exist outside of the *Methanocella* genus in other isolates of the archaeal and/or bacterial clade. Indeed, BLAST search with the GGDEF domain of GGDEF_MRE50 indicated that no other protein with the identical domain structure exists in the database. Most closely related proteins with a high identity/similarity in the GGDEF domain of up to 46.8/56.9% are of bacterial origin (Figure S4A). Those proteins were found to be of variable length with a GGDEF‐HD‐GYP or (PAS)_x_‐GGDEF‐HD‐GYP domain structure and occasionally with an alternative N‐terminal signaling domain, if any. The sensory domains deviated most significantly in their amino acid sequences (data not shown). On the other hand, (PAS)_x_‐GGDEF‐HD‐GYP domain proteins with a > 80% query cover were identified among archaeal homologs. The GGDEF domains of those proteins possess highest identity/similarity scores of 39.5/54.6% or lower (data not shown). In conclusion, the similarity among the GGDEF domains of archaeal and bacterial origin is higher than conventionally observed among GGDEF domain proteins encoded by one genome (which display conventionally 35% or lower identity (El Mouali et al. 2017; Römling et al. 2023)). This high similarity of GGDEF domains can indicate significant horizontal exchange of genetic information between members of the two domains of life in certain ecological niches with subsequent recombination events in order to combinatorially create novel cyclic di‐GMP turnover proteins.

GGDEF domain proteins GGDEF_KLK7 and GGDEF_E3 have highly similar homologs over the entire protein length in Halobacteria from the order *Halobacteriales* (MIJ0697172.1 and MIJ0066471.1 with at least 37.5/56.5% identity/similarity, NCBI database accessed upon manuscript revision September 5, 2026). In contrast, the most similar GGDEF domain from a bacterial protein encoded by a *Methylococcales* bacterium (MCK5829069.1) shows 25.0/36.79% identity/similarity to the GGDEF domains of GGDEF_KLK7 and GGDEF_E3. We conclude from these anecdotical observations that GGDEF domain similarity is not necessarily informative for domain origin beyond proteins highly similar over the entire length in closely related species or derived from similar ecological niches in phylogenetically distinct organisms. Also, additional more closely related homologs might be found upon the availability of additional archaeal genome sequences.

### 2.8 Phylogenetic Context of the Archaeal GGDEF Domains

In order to assess the broader phylogenetic context of the GGDEF proteins, specific homologs of the GGDEF domains of GGDEF_MRE50, GGDEF_E3, and GGDEF_KLK7 with the highest similarity were retrieved after BLAST search from the archaeal clade and from all non‐redundant proteins in the NCBI database. The phylogenetic analysis of the GGDEF domains showed that they are embedded in a broader phylogenetic context in Archaea and Bacteria (Figure S4B–E). However, while closely related GGDEF domain proteins are present, the sequence diversity among the 500 most similar GGDEF domains most homologous to GGDEF_MRE50, GGDEF_E3, and GGDEF_KLK7 is substantial (Figure S5).

### 2.9 Archaeal GGDEF Domains Possess Phenotypes in the *Salmonella* Rdar Biofilm and Flagella‐Mediated Motility Model

In order to assess whether any of the three GGDEF domain proteins from *M. avoryzae* MRE50, *H. salinisoli* KLK7 and *H. borinquense* E3 possess catalytic activity, we first cloned the genes in the expression vector pBAD28 to be induced from the L‐arabinose responsive pBAD promoter (Guzman et al. 1995). In order to cover the entire protein, the longest predicted open reading frame has been chosen after assessment by the Open Reading Frame Finder (ORF Finder; ncbi.nlm.nih.gov/orffinder/). Subsequently, we assessed the effect of production of the wild type gene products on rdar biofilm formation and motility in the *S. typhimurium* UMR1 (ATCC 14028 Nal^r^ rdar_28°C_) model system (El Mouali et al. 2017). The red, dry, and rough rdar biofilm model is a cyclic di‐GMP activated colony morphotype grown on agar plate. Selective uptake of the diazo dye Congo red by the two extracellular matrix components, amyloid curli and the polysaccharide cellulose, results in a characteristically colored colony with distinct surface structure and hydrophobicity. Thereby, overexpression of diguanylate cyclases stimulate rdar biofilm formation as assessed by enhanced dye uptake and colony structure (Simm et al. 2004), while cyclic di‐GMP specific phosphodiesterase activity downregulates rdar biofilm formation (Cao et al. 2026; Simm et al. 2004). In contrast, the reverse life style motility, such as flagella‐based swimming motility, is inhibited by diguanylate cyclase activity. In this context, phosphodiesterase stimulated swimming motility is most sensitively observed in the *S. typhimurium* UMR1 Δ*yhjH* mutant deleted of the phosphodiesterase dedicated for the degradation of motility‐restricting cyclic di‐GMP. Of note, these assays are not absolutely predictive on their own as for example, cyclic di‐GMP binding proteins can show phenotypic alterations reminiscent of phosphodiesterase activity, while catalytically inactive EAL domains acting through protein–protein interaction can show phenotypic alterations reverse to their catalytic counterparts (Ahmad et al. 2013; Simm et al. 2009). In combination with amino substitutions in the catalytic site, however, these and other in vivo assays allow a reliable assessment of the catalytic activity of the archaeal GGDEF and GGDEF‐HD‐GYP domain proteins.

To this end, production of the wild type protein GGDEF_MRE50 of *M. avoryzae* MRE50 in *S. typhimurium* UMR1 and UMR1 Δ*yhjH* strain did not alter the rdar biofilm morphotype (Figure 3A). As a GGDEF‐HD‐GYP hybrid protein, GGDEF_MRE50 is predicted to display diguanylate cyclase and phosphodiesterase activity. Thus, GGDEF_MRE50 might simultaneously express both activities, which might nullify the phenotype. Consequently, we subsequently assessed the effect of a protein truncated of its C‐terminal HD‐GYP domain, which again did not alter the rdar morphotype. Further, we abolished the phosphodiesterase and diguanylate cyclase activity by introducing the D551A/E552A amino acid substitutions in the GGD_551_E_552_F motif of the GGDEF domain and the D680A amino acid substitution in the HD_680_ motif of the HD‐GYP domain. Production of these mutant proteins did, however, again not alter rdar biofilm formation compared to the *S. typhimurium* UMR1 vector control. In contrast, upregulated motility upon production of GGDEF_MRE50 and its GGAAF catalytic mutant in *S. typhimurium* UMR1 Δ*yhjH* was observed (Figure 3B). In agreement, the HD‐GYP catalytic mutant of GGDEF‐MRE50 and the mutant deleted for the HD‐GYP domain showed vector control motility, indicating that the HD‐GYP domain is a cyclic di‐GMP phosphodiesterase, while diguanylate cyclase activity of GGDEF_MRE50 has not been observed in this context.

**FIGURE 3:**
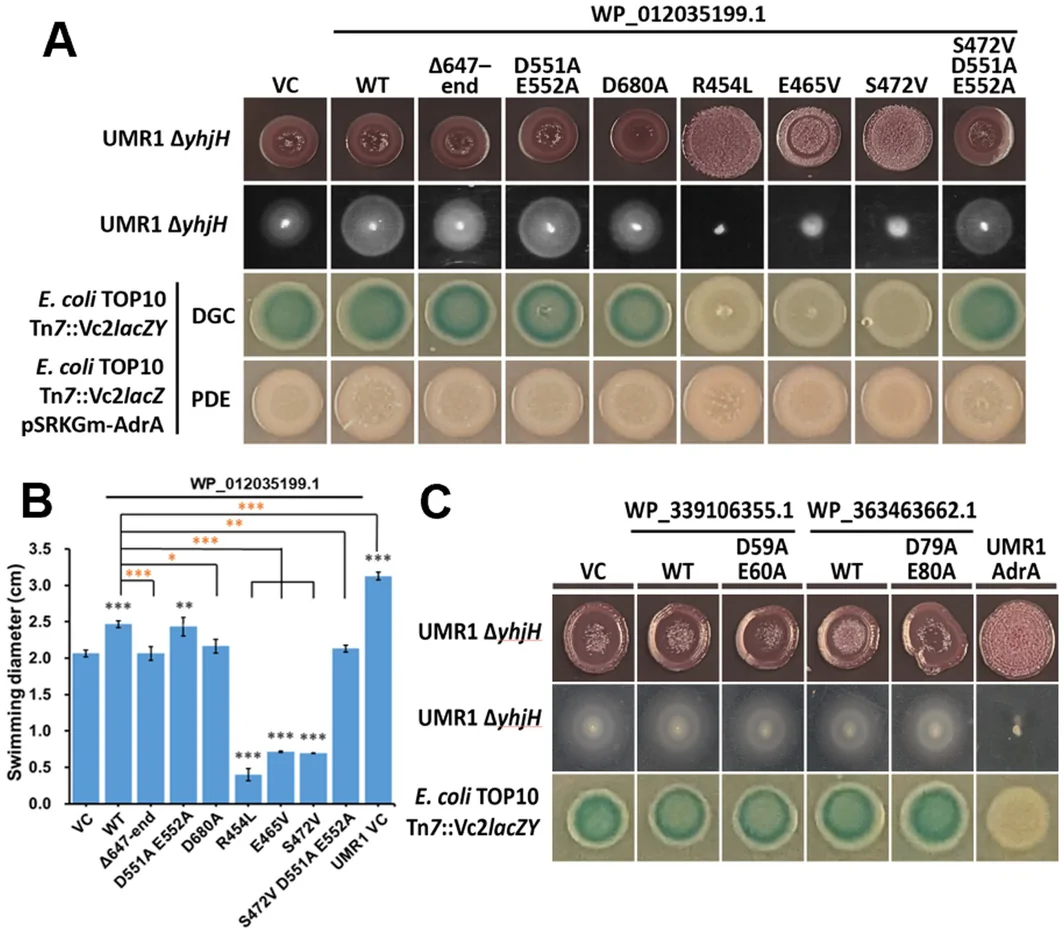
In vivo assays rdar biofilm formation and flagella based swimming motility in the *S. typhimurium* UMR1 model and Vc2 riboswitch assay in *E. coli* TOP10 Tn*7*::Vc2*lacZY* to assess catalytic activity of the GGDEF‐HD‐GYP domain protein GGDEF_MRE50 (WP_012035199.1) from *M. arvoryzae* MRE50 and its variants under different growth conditions. (A) Rdar colony morphology assay to assess the catalytic activity of GGDEF_MRE50 from *M. arvoryzae* MRE50 and its variants in *S. typhimurium* UMR1 Δ*yhjH* on CR agar plates using LB without NaCl medium (first row). Ten μL of a bacterial suspension of OD_600_ = 5 were placed on the agar, dried and subsequently incubated at 28°C. Proteins were produced under the control of the pBAD promoter induced with 0.1% L‐arabinose and development of colony morphology and color assessed up to 72 h. Displayed is rdar biofilm development after 48 h. Scale: width of individual figure corresponds to 14 mm. Flagella based swimming motility assay to assess the catalytic activity of GGDEF_MRE50 from *M. arvoryzae* MRE50 and its variants in *S. typhimurium* UMR1 Δ*yhjH* (second row). Two μl of a bacterial suspension of OD_600_ = 5 were inoculated into the 0.25% agar with LB as medium and the plate subsequently incubated at 37°C. Motility was documented after 6 h. Proteins were produced under the control of the pBAD promoter induced with 0.1% L‐arabinose. MAE108 ( *S. typhimurium* UMR1 Δ*fliC* Δ*fljB*) served as a negative control. Scale: Width of individual figure corresponds to 36 mm. Vc2‐based riboswitch assay to assess diguanylate cyclase catalytic activity of GGDEF and GGDEF‐HD‐GYP domain proteins. Catalytic activity of GGDEF_MRE50 from *M. arvoryzae* MRE50 and its variants was assessed in *E. coli* TOP10 Tn*7*::Vc2*lacZY* via downregulation of β‐galactosidase activity (third row). Three μl of a bacterial suspension of OD_600_ = 5 were placed onto an LB agar plate containing 80 μg mL^−1^ X‐gal and appropriate antibiotics. Plates were incubated up to 72 h to assess diminished development of the blue precipitate 5,5′‐di‐bromo‐4,4′‐di‐chloroindigo obtained after dimerisation and oxidation of the enzymatic product 5‐bromo‐4‐chloro‐3‐hydroxyindole. Scale: width of individual figure corresponds to 7 mm. Cyclic di‐nucleotide controls for cyclic di‐GMP were *E. coli* TOP10 Tn*7*::Vc2*lacZY* pAdrA and the pAdrA G288A, G289A mutant; pDncV and its catalytic pDncV D129A, D131A mutant and pCdaS and its catalytic pCdaS D135A mutant (in Figure S6). Vc2‐based riboswitch assay to assess phosphodiesterase catalytic activity of EAL and HD‐GYP domain proteins (forth row). Catalytic activity of GGDEF_MRE50 from *M. arvoryzae* MRE50 and its variants was assessed in *E. coli* TOP10 Tn*7*::Vc2*lacZY* pAdrAGm by upregulation of β‐galactosidase activity. Three μl of a bacterial suspension of OD_600_ = 5 were placed onto an LB agar plate containing 80 μg mL^−1^ X‐gal and appropriate antibiotics. Plates were incubated up to 72 h to assess development of 5,5′‐dibromo‐4,4′‐dichloroindigo after dimerisation and oxidation of the product of the enzymatic activity. Cyclic di‐nucleotide controls for cyclic di‐GMP hydrolysis were pYhjH and its pYhjH E48A and pYhjH E136A catalytic mutants (in Figure S6). (B) Quantitative estimation of flagella based swimming motility to assess the catalytic activity of GGDEF_MRE50 from *M. arvoryzae* MRE50 and its variants in *S. typhimurium* UMR1 Δ*yhjH*. p* < 0.05, p** < 0.01, p*** < 0.001 by student's *t*‐test compared to *S. typhimurium* UMR1 Δ*yhH* vector control pBAD28 (black stars) and *S. typhimurium* UMR1 Δ*yhH* with GGDEF_MRE50 cloned in pBAD28 (red stars). (C) In vivo assays rdar biofilm formation and flagella based swimming motility in the *S. typhimurium* UMR1 model and Vc2 riboswitch based expression of LacZY in *E. coli* TOP10 Tn*7*::Vc2*lacZY* to assess catalytic activity of the GQGDEF domain proteins GGDEF_KLK7 from *H. salinisoli* KLK7 and GGDEF_E3 from *H. borinquense* E3 and its catalytic GQGAAF motif mutants under different growth conditions. Assays were conducted as described for Figure 3A besides that L‐arabinose has been omitted in the rdar biofilm assay and flagella‐based motility was documented after 5 h. Scale: width of individual figure corresponds to 14, 35, and 7 mm for rdar biofilm, swimming motility and riboswitch assay, respectively.

### 2.10 Linker Mutations Trigger Diguanylate Activity of the GGDEF_MRE50 GGDEF Domain

Phenotypic assessment upon expression of the complex GGDEF_MRE50 GGDEF‐HD‐GYP domain protein of *M. avoryzae* MRE50 was only informative with respect to the phosphodiesterase activity of the HD‐GYP domain in the motility assay (Figure 3A). At this state of investigation, it cannot be excluded that the observed cyclic di‐GMP dependent phosphodiesterase activity is a minor catalytic activity. Proof of diguanylate cyclase activity of the GGDEF domain of GGDEF_MRE50 will ultimately indicate synthesis of cyclic di‐GMP by this archaeal protein. Consequently, we aimed to identify activating signals or, alternatively, design a protein variant that displays substantial diguanylate activity by its GGDEF domain.

First, we inspected the three signaling domains (Figure 1B). Most obviously, phosphorylation of the N‐terminal REC_S_ receiver domain on the conserved aspartate at position equivalent 56 could affect the activity of downstream catalytic domains. To this end, the D58E mutant mimicking phosphorylation did not affect the behavior of GGDEF_MRE50 (data not shown). The signals that are sensed by the PAS/PAC and PocR domains of GGDEF_MRE50 are currently unknown. A structural model created by AlphaFold 3 showed that full‐length GGDEF_MRE50 formed a non‐productive dimer with the GGDEF domains located at opposite sites (Figure S5). We concluded that the input of multiple signals might be required to stimulate the catalytic activity of the GGDEF domain of GGDEF_MRE50.

Signaling and catalytic domains are conventionally connected by linkers that can display an α‐helical secondary structure designated as signaling S‐helix which conditional form a leucine zipper (Anantharaman et al. 2006). To allow flexible and reversible conformational states these linkers possess amino acid motifs predominantly incomplete for constitutive dimerization. Linkers can, however, reversibly dimerize upon allosteric transmission of the signal via coiled‐coil interactions and switch between a repressive and active register dependent on the signal transduction mediated allosteric conformation changes (Baral et al. 2025; De et al. 2009; Gourinchas et al. 2017). Naturally or engineered, linkers can exist as constitutive dimerization modules (Baral et al. 2025; De et al. 2009; Gourinchas et al. 2017; O'Shea et al. 1991; Rasmussen et al. 1991).

We noted that the 57 aa long α‐helical linker which connects the third N‐terminal signaling domain PocR with the GGDEF domain includes the sequence of an incomplete activating leucine zipper heptad repeat (Figures 1B and 4; (Schirmer 2016)). When the N‐terminal REC_S_, PAS/PAC and PocR domains were deleted, AlphaFold 3 modelling predicted a protein dimer with GGDEF domains facing each other with their active sites in a catalytically competent arrangement (Figure 4C). The linker sequence of GGDEF_MRE50 deviates from the sequence that can provide effective constitutive dimerization leading to constitutively active downstream domains (Figure 4B). Consequently, we introduced single amino acid substitutions designed to provide tight interactions between the two α‐helices of the linker dimer in a productive configuration and assessed their effects on the performance of the enzyme. To this end, we constructed R454L, E465V, and S472V substitutions. Surprisingly, while the R454L mutant had the most pronounced effect, any of the single amino acid substitutions significantly triggered rdar biofilm formation in *S. typhimurium* UMR1 (Figure 3A). Of note, the motility assay did not show diminished motility by any of the linker mutants in the *S. typhimurium* UMR1 strain. Expression of linker mutants, however, substantially downregulated motility in *S. typhimurium* UMR1 Δ*yhjH* indicating apparent diguanylate cyclase activity (Figure 3A,B). These observations indicated that cyclic di‐GMP is produced by GGDEF_MRE50, but the amount is not sufficient to overcome the motility‐dedicated phosphodiesterase activity of YhjH. Deletion of *yhjH*, however, allowed inhibition of motility by GGDEF_MRE50 produced cyclic di‐GMP. Combination of the S472V linker mutant with the D551A/E552A catalytic site mutant of the GGDEF domain displayed a rdar morphotype and motility not significantly different from the vector control and HD‐GYP mutants respectively. These results demonstrated that an optimized linker configuration does not only trigger diguanylate cyclase activity by productive GGDEF domain dimerization, but also inhibits HD‐GYP dependent phosphodiesterase activity of GGDEF_MRE50 (Figure 3). In conclusion, directed mutations promoting productive dimerization of the linker connecting the PocR signaling domain with the GGDEF domain enabled pronounced diguanylate cyclase activity of GGDEF_MRE50. It might be of interest to assess the effect of mutations in the other linkers of GGDEF_MRE50.

**FIGURE 4:**
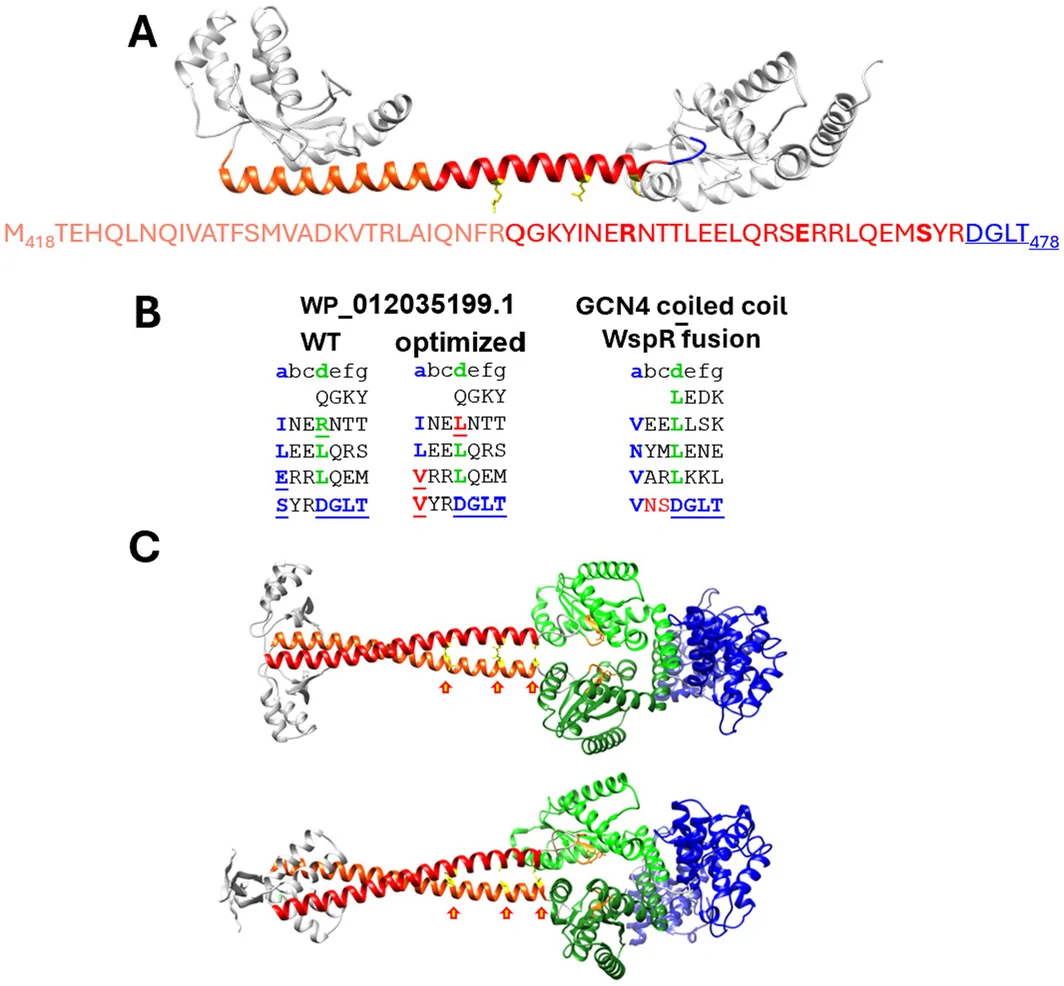
Characterisation of the linker region between the PocR and the GGDEF domain of the GGDEF_MRE50 protein (WP_012035199.1) from *M. arvoryzae* MRE50 as a hydrophobic heptad repeat sequence potentially capable to form coiled‐coil interactions. (A) AlphaFold 3 structural model of the linker (in orange and red) with the wide‐turn at the C‐terminal end containing the DGLT motif (in blue). The linker is flanked by the PocR and the GGDEF domain (in grey), respectively. Positions a and d comprise amino acids with an aliphatic side chain such as valine and leucine. The amino acids to be substituted for an optimal heptad repeat sequence are indicated with their side chains in yellow. Amino acid sequence of the linker between the PocR and the GGDEF domain with relevant amino acids required for coiled coil structure formation and amino acids to be altered in bold. (B) Left, GGDEF_MRE50 wild type heptad repeat sequence of the linker region ending C‐terminal at the DGLT motif which marks the wide turn at the start of the GGDEF domain compared to an optimized sequence dimerizing as a productive heptad repeat linker (middle) and the GCN4‐WspR_GGDEF_ hybrid linker sequence (right). (C) Dimer formation of the wild type linker‐GGDEF‐HD‐GYP domain protein GGDEF_MRE50 from *M. arvoryzae* MRE50 (upper structural model) and the three amino acid substitution linker variant (lower panel). The amino acids to be substituted for an optimal heptad repeat sequence and substituted are indicated with their side chains in yellow and by arrows. The GGDEF domains of the monomer face each other in an apparently active constellation. Linker in red, GGDEF domain in green, HD‐GYP domain in blue and the β‐hairpin with the GGDEF motif in orange.

### 2.11 Archaeal GGDEF Domain Protein Show Diguanylate Cyclase Activity in an In Vivo Riboswitch Assay

Rdar biofilm and motility assays of GGDEF_MRE50 wild type protein in combination with catalytic mutants indicated that the observed phenotypic alterations are due to the catalytic activity of both domains associated with cyclic di‐GMP turnover. In order to verify whether indeed alterations in the concentration of cyclic di‐GMP are responsible for the phenotypic alterations we used a previously developed detection assay based on the Vc2 riboswitch located in single copy in the Tn*7* site in *E. coli* TOP10 (El Mouali et al. 2017; Liu et al. 2018; Liu et al. 2020). This assay is based on the alteration of β‐galactosidase activity in response to alternating concentrations of cyclic di‐GMP monitored by accumulation of a chromogenic product. Upon binding with nanomolecular affinity to the Vc2 aptamer to cause a conformational change in the riboswitch, cyclic di‐GMP inhibits the translation of the downstream gene product (Sudarsan et al. 2008). Diguanylate cyclase activity can be readily and sensitively monitored, resulting in significant downregulation of the β‐galactosidase activity (El Mouali et al. 2017; Liu et al. 2018; Liu et al. 2020). To this end, this riboswitch assay indicated no cyclic di‐GMP turnover in the wild type, the HD‐GYP domain deletion mutant and all the catalytic mutants of wild type GGDEF_MRE50 (Figure 3C). Production of the three linker mutants, however, indicated significant diguanylate cyclase activity as β‐galactosidase activity was abolished. Recovery of β‐galactosidase activity upon the introduction of the D551A/E552A mutation in the GGDEF motif in the S472V linker mutant suggested loss of the catalytic activity. These observations are consistent with the biofilm and motility assays and demonstrate diguanylate cyclase activity of the GGDEF domain of GGDEF_MRE50.

Assessment of phosphodiesterase activity is most sensitively and consistently monitored in the background of elevated cyclic di‐GMP concentrations produced by a diguanylate cyclase (El Mouali et al. 2017). As previously engineered constructs expressing the diguanylate cyclase AdrA were incompatible with expression of the GGDEF_MRE50 open reading frame under the L‐arabinose inducible pBAD promoter, we cloned AdrA in the pSRKGm vector under the control of the leaky p*lac* promoter. To this end, this riboswitch assay indicated no cyclic di‐GMP degradation by the wild type, the HD‐GYP deletion mutant, and the catalytic mutant proteins (Figure 3D). The linker mutant proteins, however, downregulated the β‐galactosidase activity even more, indicating diguanylate cyclase activity which was relieved by the introduction of the D551A/E552A mutation in the GGDEF motif.

Potential catalytic activities of GGDEF_KLK7 and GGDEF_E3 were monitored in biofilm, motility, and Vc2 riboswitch assays (Figure 3A–D). To this end, production of the two proteins and their GQGAAF mutants showed upregulation of rdar biofilm formation in the *S. typhimurium* UMR1 Δ*yhjH* mutant without arabinose induction as the only visible outcome. Thus, while heterologous expression indicated physiological activity, no indications for catalytic activity could be collected.

In conclusion, the Vc2::*lacZY* riboswitch assay indicated diguanylate cyclase activity of the GGDEF domain of GGDEF_MRE50 in the linker mutant background consistent with the rdar biofilm and motility assays.

### 2.12 Selected GGDEF Domain Proteins From MAGs Encode Functional Diguanylate Cyclases

Having demonstrated that the archaeal isolate *M. avoryzae* MRE50 encodes an enzymatically functional GGDEF domain protein, we were wondering whether GGDEF domains with diguanylate cyclase activity are more widespread in Archaea. To this end, we assessed the phylogenetic origin of the contigs of previously retrieved archaeal candidate GGDEF protein by investigating at least two additional gene products flanking the GGDEF protein gene up‐ and down‐stream, respectively. Proteins were only selected when their genes were flanked by open reading frames of unambiguous archaeal origin. We subsequently selected 20 GGDEF domain proteins to be encoded mainly by archaeal contigs derived from the DPANN and class II archaeal clades (Figure 5, Figure S6). Several of these proteins were either GGDEF only proteins or proteins with an N‐terminal or C‐terminal amino acid sequence too short to comprise a domain (Figure 5A). Short GGDEF domain only proteins are rarely found in Bacteria. Diverse N‐terminal signaling domains such as a GAF, REC or PAS/PAC domain, a PocR‐GAF domain combination and a 7TM domain were N‐terminal of other GGDEF domain proteins. All those signaling domains have also been identified in bacterial GGDEF domain proteins with PAS/PAC, GAF and REC domains being most frequent (Anantharaman and Aravind 2005; Xing et al. 2023). One protein, RJQ22058.1, contained an undefined short domain at the N‐terminus. Alignment of the GGDEF domains of all those proteins with class I, II and III GGDEF domains indicated that the majority of these proteins had the GG(D/E)EF motif and additional signature amino acid required for catalytic activity conserved (Figure S7). Three of the proteins showed unconventional GGDEF motifs or insertions close to the central catalytic GGDEF motif. These were MBI503694.1 with a RIFHPSGDEF motif, UCD02967.1 with a RYGGDKGDEF and MBN1216112.1 with a 12 amino acid long insertion upstream of the RxxGGDEF motif. None of the 20 GGDEF domain proteins contained the signature serine replacing the aspartate in the xxGD motif indicative for cyclic GMP‐AMP activity of a GGDEF domain as in MXAN_2643 and GSU1658 (Hallberg et al. 2019). The structural models of all GGDEF domains were most similar to crystal structures of catalytically active GGDEF domains (Figure 5B,C and data not shown). Subsequent construction of a phylogenetic tree of the 20 GGDEF domains with experimentally confirmed GGDEF domain proteins showed a cluster of archaeal GGDEF domains closest to functional WspR from *P. aeruginosa* and AdrA of *S. typhimurium* . Other archaeal GGDEF domains are, however, also found outside of this cluster, dispersed throughout this phylogenetic tree with diverse reference GGDEF domains (Figure 5D). In conclusion, a diversity of GGDEF domain proteins with potential diguanylate cyclase activity can also be found in MAGs validated to originate from Archaea.

**FIGURE 5:**
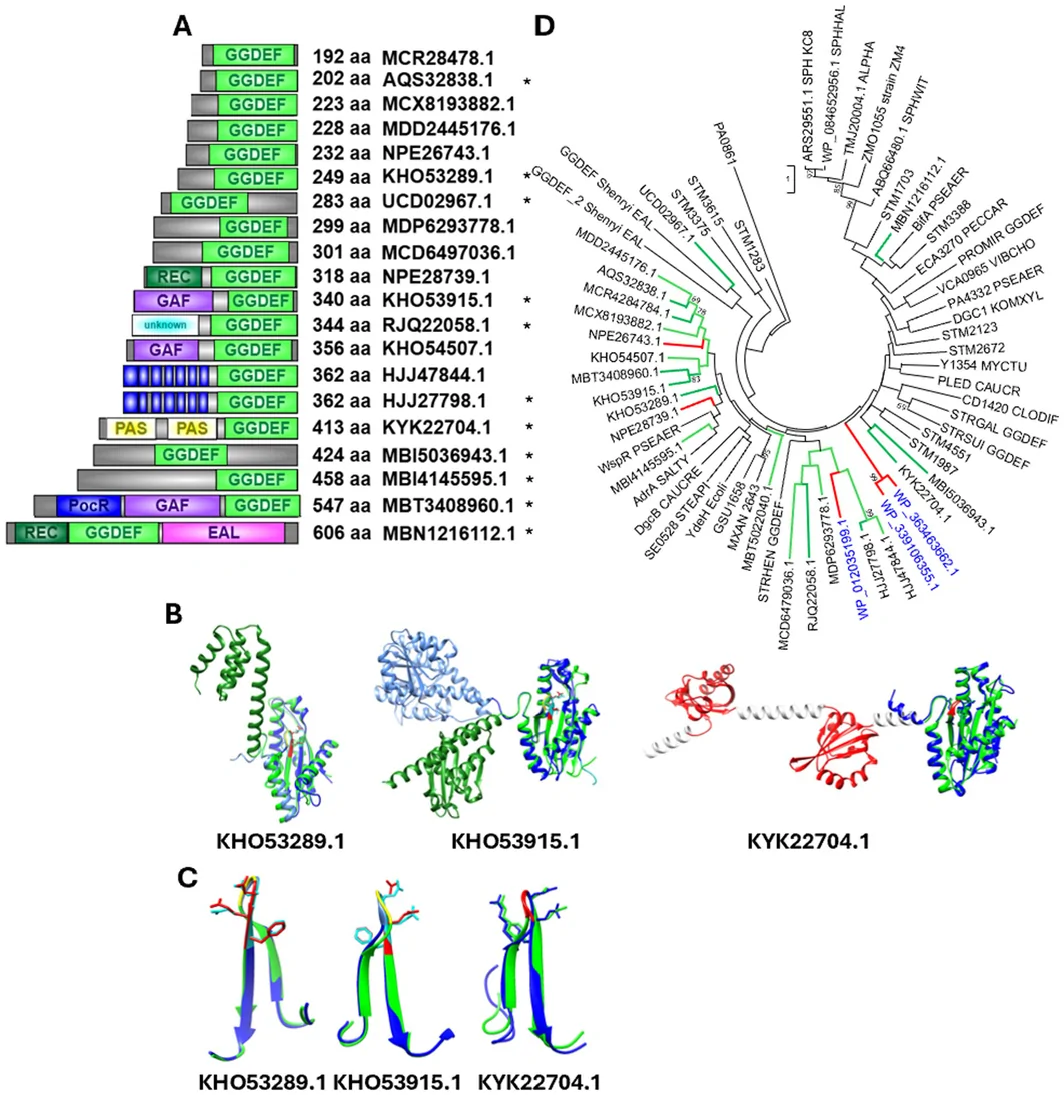
Characterisation of the 20 archaeal GGDEF domain proteins considered for analysis. (A) Domain structure of the 20 selected GGDEF domain proteins from archaeal MAGs. Domain structures were drawn with IBS 2.0 (Xie et al. 2022). The 11 proteins eventually cloned are indicated by an asterix. (B) Overlays of AlphaFold 3 structural models of GGDEF domain proteins KHO53289.1, KHO53915.1, and KYK22704.1 with the most similar experimentally determined GGDEF domain structures as screened with FoldSeek. (C) Close‐up of the overlays of the β‐hairpins with the GGEEF motif. The β‐hairpins of archaeal GGDEF domains are highly congruent with the experimentally determined structures of *Marinobacter aquaeolei* VT8 (PDB: 3IGN), the activated diguanylate cyclase PleD of *C. vibrioides* (PDB: 2WB4) and the LOV‐activated dark‐site diguanylate cyclase of *Methylotenera* sp. (PDB: 8c05), respectively, in the limit of the resolution of the respective crystal structure. (D) Phylogenetic comparison of the GGDEF domains of the 20 archaeal proteins from MAGs and the three GGDEF domain proteins from archaeal isolates with GGDEF domains from experimentally analyzed proteins. The identity of the GGDEF domain proteins is indicated in the Supporting Information. After alignment with ClustalX 2.1 (Higgins and Sharp 1988) and manual curation in GeneDoc, phylogenetic assessment was performed in MEGA 7.0 (Kumar et al. 2016) using the Maximum Likelihood approach. Statistical significance of the phylogenetic tree branches was assessed with 100 bootstraps. The alignment of the GGDEF domains of selected candidate proteins from archaeal MAGs for further analysis is shown in Figure S7.

### 2.13 Phenotypic Assays for Archaeal GGDEF Proteins From MAGs Indicate Catalytic Activity

To this end, we cloned seven selected GGDEF domain proteins and constructed their GGAEF catalytic mutants in the GG(D/E)EF motif to assess their capability to synthesize cyclic di‐GMP by the rdar biofilm, motility and the Vc2 riboswitch assay. We found indications for catalytic activity of GGDEF proteins in all three assays; however, none of the proteins consistently showed indications for catalytic functionality in the three assays (Figure 6, Figure S8). For example, the *E. coli* TOP10 Tn*7*::Vc2*lacZY* riboswitch assay indicated diguanylate cyclase activity for KOH53289.1 and MBN1216112.1 as relief of the downregulation of the β‐galactosidase activity by wild type protein occurred in the respective GGAEF mutant. However, the other two assays were not informative for diguanylate cyclase activity for these proteins. Indeed, a reverse motility behavior is observed for KOH53289.1 and its catalytic mutant. In contrast, closely related KOH53915.1 and its catalytic mutant showed a conventional behavior in the motility and *E. coli* TOP10 Tn*7*::Vc2*lacZY* riboswitch, but not in the biofilm assay. On the other hand, production of KY476720.1, MBN1216112.1, and MBT3408960.1 showed upregulated biofilm behavior either in *S. typhimurium* UMR1 upon 0.1% L‐arabinose induction or in *S. typhimurium* UMR1 Δ*yhjH* without L‐arabinose, respectively, independently of catalytic activity. In conclusion, none of the proteins consistently and robustly showed indication of diguanylate cyclase activity in all three assays. These results indicate that catalytic activity is highly dependent on the growth conditions, the genetic background and/or the bacterial species. The riboswitch assay has been proven to be most sensitive and unambiguous with indication of diguanylate cyclase activity for three of seven of the investigated GGDEF domain proteins. As the Vc2 aptamer displays a 1 nM affinity for cyclic di‐GMP (Sudarsan et al. 2008), scaffold and protein–protein interaction effects are not expected to grossly interfere with the sensitive monitoring of alterations in cyclic di‐GMP levels.

**FIGURE 6:**
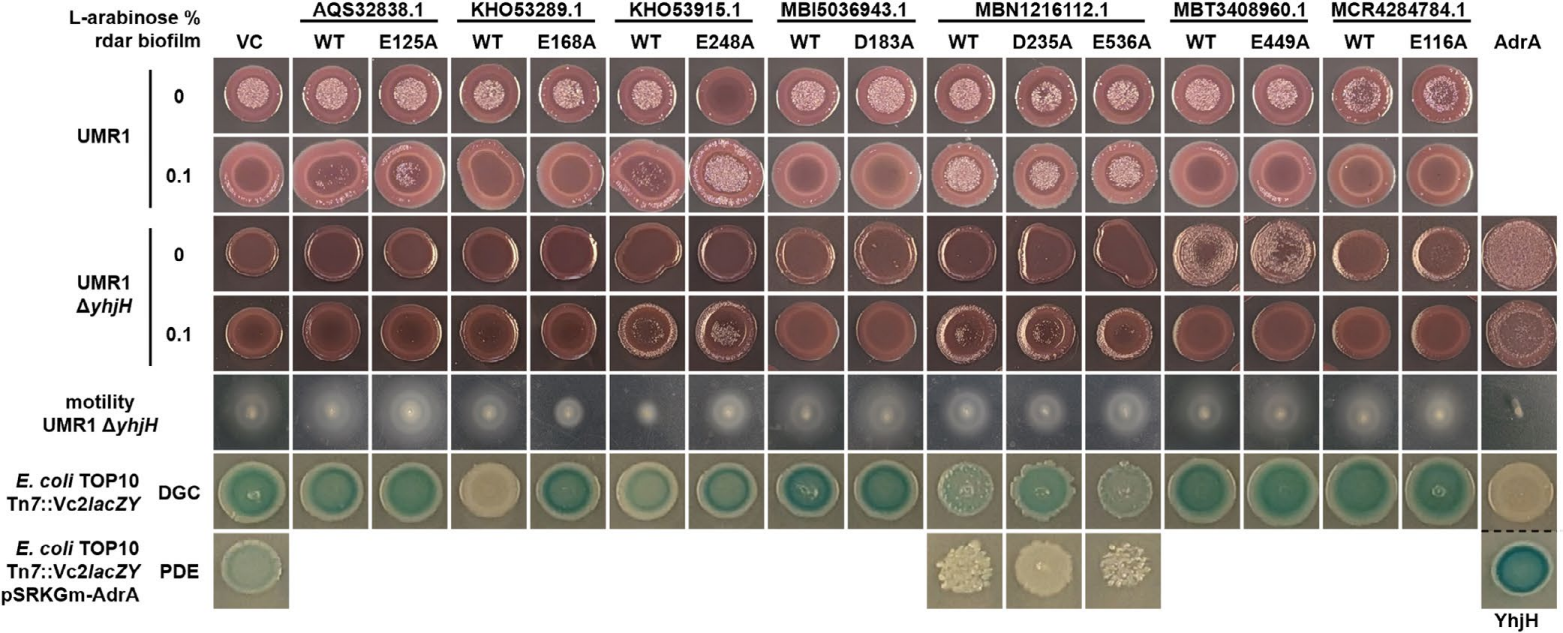
In vivo assays rdar biofilm formation and flagella based swimming motility in the *S. typhimurium* UMR1 and UMR1 Δ*yhjH* model and Vc2 riboswitch assay in *E. coli* TOP10 Tn*7*::Vc2*lacZY* to assess the catalytic activity of GGDEF domain proteins from metagenome‐assembled genomes and its catalytic variants. The genes and variants had been cloned into pBAD28 vector. Assay conditions as described in Figure 3C. Biofilm formation has been assessed without and with 0.1% L‐arabinose induction. Scale: width of individual figure corresponds to 14, 35, and 7 mm for rdar biofilm, swimming motility and riboswitch assay, respectively.

### 2.14 The PAS‐PAS‐GGDEF Domain Protein KYK2270.1 Possesses Pronounced Diguanylate Cyclase Activity in the *S. typhimurium* Rdar Biofilm and Motility Model

As none of the previously MAG‐derived GGDEF domain proteins consistently indicated catalytic activity in all three in vivo assays, we investigated four additional GGDEF domain proteins, KYK22704.1, UCD02967.1, HJJ27798.1, and RJQ22058.1 (Figure 5A). To this end robust diguanylate cyclase activity was observed for the PAS‐PAS‐GGDEF protein KYK22704.1 compared to its GGAAF catalytic variant in the biofilm and motility assays, equally as in the *E. coli* TOP10 Tn*7*::Vc2*lacZY* riboswitch assay (Figure 7). As the assays indicated pronounced diguanylate cyclase activity, we assessed the catalytic activity of the enzyme at the elevated temperatures of 37°C and 42°C. Indeed, catalytic activity, as indicated by elevated rdar biofilm formation upon expression of the wild type protein, but not its GGAAF variant, was observed. As KYK22704.1 was highly active, we assessed the role of the RxxD motif located six amino acids N‐terminal of the GGDEF motif, which is conventionally part of the allosteric inhibitory (I)‐site (Christen et al. 2006). While the R330A mutant showed extended enhanced rdar biofilm formation compared to the wild type protein, as expected from the impairment of the I‐site, the D333A mutant displayed enhanced rdar morphotype development only at 28°C. In contrast, a downregulated rdar morphotype was displayed at 37°C, indicating reduced apparent catalytic activity.

**FIGURE 7:**
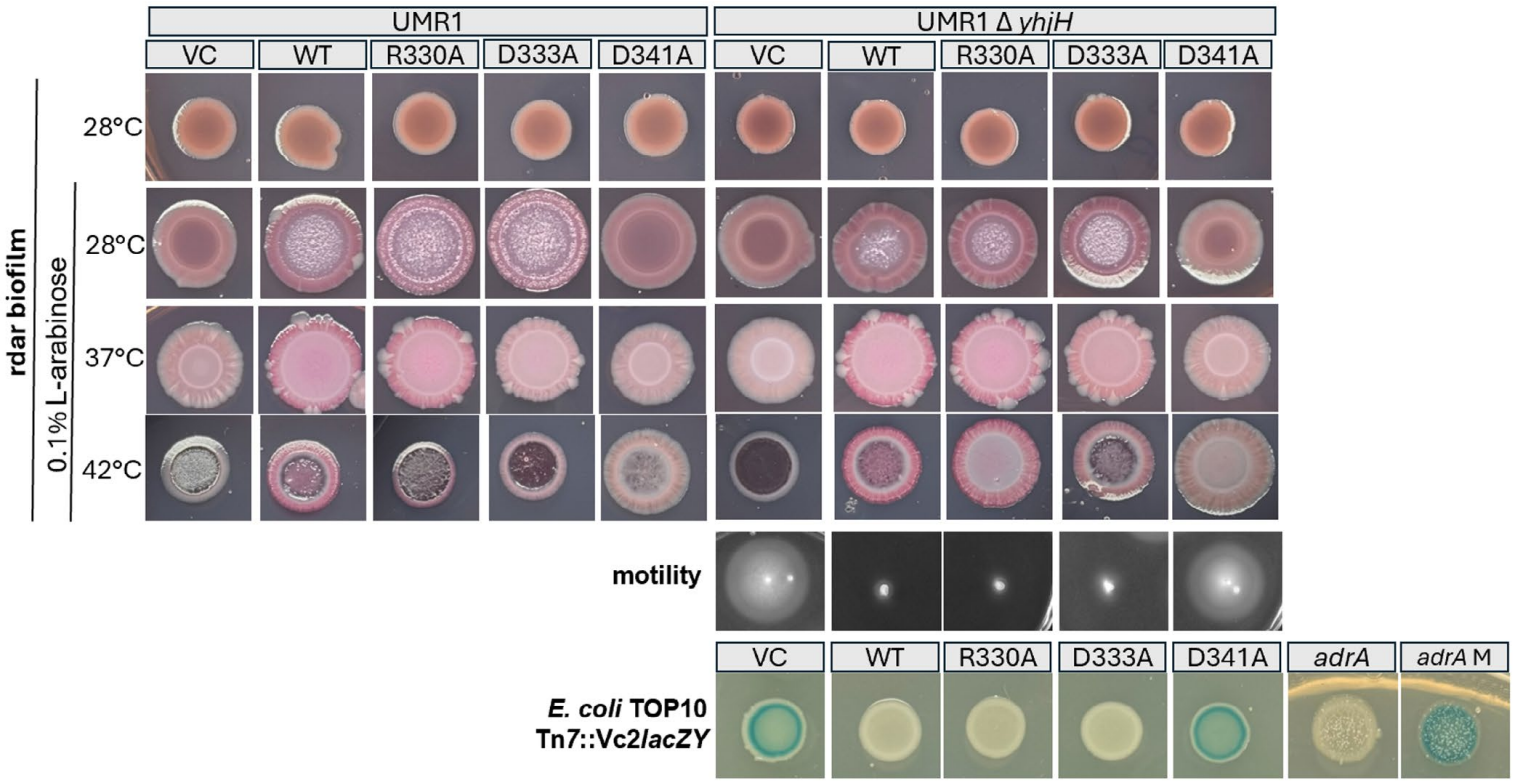
In vivo assays rdar biofilm formation and flagella based swimming motility in the *S. typhimurium* UMR1 and UMR1 Δ*yhjH* model and LacZY activity regulated by the Vc2 riboswitch in *E. coli* TOP10 Tn*7*::Vc2*lacZY* to assess the catalytic activity of the PAS‐PAS‐GGDEF domain protein KYK22704.1 from a metagenome‐assembled genome, its catalytic variant with the GGAAF motif and alanine substitutions in the RxxD motif which is part of the allosteric inhibitory I‐site. Assay conditions as described in Figure 3C. The gene and variants had been cloned into the pBAD28 vector. Biofilm formation has been assessed without and with 0.1% L‐arabinose induction. Scale: width of individual figure corresponds to 14, 35, and 7 mm for rdar biofilm, swimming motility and the riboswitch assay, respectively.

Among the other selected MAG derived GGDEF proteins UCD02967.1, HJJ27798.1, and RJQ22058.1 production of RJQ22058.1 upregulated biofilm formation, repressed motility and β‐galactosidase activity in the riboswitch assay while the D256A catalytic mutant acted as the vector control indicating diguanylate cyclase activity of RJQ22058.1 in the *Salmonella* and *E. coli* models (Figure S9). In contrast, production of UCD02967.1 and HJJ27798.1 and its catalytic mutants did not significantly alter the phenotypes compared to the vector control.

In conclusion, KYK22704.1 and RJQ22058.1 are prime candidates for more detailed molecular and biochemical investigations as all three in vivo assays consistently indicated diguanylate cyclase activity of these GGDEF domain proteins.

## 3 Discussion

### 3.1 GGDEF Domain Diguanylate Cyclases Are Found in Different Archaeal Branches

Cyclic di‐GMP is a multifunctional nearly ubiquitous second messenger in Gram‐negative and Gram‐positive Bacteria regulating the fundamental single cell life style switch between motility and sessility (Purcell and Tamayo 2016; Römling et al. 2013). In this work we provided evidence for cyclic di‐GMP as a primordial molecule dedicated to the regulation of biofilm formation at the origin of life already in LUCA or even already in the first universal common ancestor (FUCA). We show that GGDEF domain proteins are encoded and expressed by Archaea and predominantly produce the second messenger cyclic di‐GMP. These findings indicate cyclic di‐GMP and presumably an entire signaling cascade including cyclic di‐GMP specific phosphodiesterases and receptors to be indeed present in multiple branches of Archaea supporting the hypothesis of an ancient origin of this nearly ubiquitous second messenger molecule.

Importantly, we identified GGDEF domain proteins that synthesize cyclic di‐GMP in archaeal isolates from different branches of the archaeal phylogenetic tree, isolates *M. arvoryzae* MRE50, *H. salinisoli* KLK7 and *H. borinquense* E3, and *Methanococcoides* sp. SA1 (NCBI accession number JABKBV010000000, not experimentally investigated). Indeed, the *Methanococcoides* sp. SA1 genome even codes for two GGDEF domain proteins (Figure 5, Figure S7). As a broader perspective, this work shows that GGDEF domain proteins from Archaea show biological, catalytic and catalytically independent scaffold‐based activities in the *S. typhimurium* and *E. coli* model systems, indicating a universal functionality and promiscuity of the cyclic di‐GMP signaling system. In the case of phenotypes not mediated by the catalytic activity of the enzymes and of novel phenotypes mediated by catalytically competent proteins, the model systems can serve to assess novel regulatory pathways and protein–protein interactions that support the universal functionality and exceptional modularity of this signaling system.

### 3.2 The Phylogenetic Origin of Archaeal GGDEF Domain Diguanylate Cyclases Needs to be Investigated

While we show in this work that cyclic di‐GMP signaling is indeed present in Archaea, open questions remain. First, we have not systematically investigated the presence of GGDEF domain proteins throughout the archaeal domain of life. Such a task might not even be possible considering the sparsity of archaeal genome sequences. Thus the fraction of archaeal isolates that possess a GGDEF domain protein and potentially a cyclic di‐GMP signaling network is currently unknown. However, our preliminary overall assessment of the occurrence of GGDEF domains in Archaea indicates that GGDEF domain mediated cyclic di‐GMP signaling might only occasionally be found in this domain of life. In contrast to Bacteria, preliminary evidence indicates that turnover proteins are not found in multiple copies encoded by one chromosome. However, a scarce occurrence might not necessarily be an indication of horizontal gene transfer from Bacteria at some point in evolution as cyclic di‐GMP signaling and its synthesizing and hydrolyzing proteins can rapidly disappear on an evolutionary time scale (Liu et al. 2020). Verified reasons for the rapid disappearance of cyclic di‐GMP are niche restriction such as in *Shigella* spp., genome reduction and the use of cyclic di‐GMP as a molecule mediating phage defense (Lai et al. 2009; Morehouse et al. 2020; Ojha et al. 2021). Although sessility/motility dedicated cyclic di‐GMP signaling is (nearly) ubiquitous in Bacteria, it occurs rarely in distinct phyla such as *Bacteroidota* due to interference with phage defense signaling (Morehouse et al. 2020). In addition, although cyclic di‐GMP signaling is predominantly present in *γ‐proteobacteria* with turnover proteins present in multiple copies (> 60 copies of GGDEF domain proteins can be encoded by one chromosome), cyclic di‐GMP signaling is (nearly) absent in the family *Morganellaceae*. This is remarkable as the family *Morganellaceae* contains many species, for example in the *Proteus* genus, that are free living organisms with a large genome size thriving in multiple ecological niches (Liu et al. 2020). On the other hand, there are also examples of regain of a cyclic di‐GMP signaling system. In certain *Streptococcus* spp., an entire signaling module comprising a diguanylate cyclase and a corresponding effector cellulose synthase like enzyme has been acquired by horizontal gene transfer (Liu et al. 2020). Thus, although the cyclic di‐GMP signaling system might have been originally present in Archaea, the possibility exists that it could have been (nearly) depleted during evolution only being maintained occasionally. In this context, GGDEF domain based cyclic di‐GMP signaling might have interfered with frequently occurring antiphage defense systems such as the cyclic‐oligonucleotide‐based anti‐phage signaling system (CBASS) (Morehouse et al. 2020). The diverse cGAS/DncV‐like nucleotidyltransferases (CD‐NTases)/SMODS nucleotide synthases of the CBASS system (which are structurally unrelated to the GGDEF domain) synthesize a number of different cyclic di‐nucleotides among them cyclic di‐GMP. However, whether CBASS CD‐NTases present in Archaea synthesize cyclic di‐GMP as part of anti‐phage defense systems and whether this occurs mutually exclusive with GGDEF based cyclic di‐GMP signaling is currently unknown.

On the other hand, extensive horizontal gene transfer has occurred and is occurring between Bacteria and Archaea that even determinatively contributed to the emerging archaeal order Halobacterales (Gophna and Altman‐Price 2022). Thus it cannot be excluded that concomitantly horizontal transfer of cyclic di‐GMP turnover protein genes has occurred that contributed to the occurrence of GGDEF domain proteins in archaeal *Halobacteria*. If so, GGDEF domain proteins were maintained as they provided a competitive advantage for the organisms. It can therefore be hypothesized that the horizontally transferred GGDEF domain proteins, perhaps together with their receptors, have opened new opportunities for forming biofilms, regulating motility or affecting other physiological features and metabolic pathways in Archaea (Liu et al. 2020; Mikkelsen et al. 2009).

### 3.3 Perspectives

In conclusion, this work opens the way to analyze cyclic di‐GMP signaling in Archaea. Immediate follow‐up questions at the level of individual genomes include the (co)occurrence of EAL and/or HD‐GYP domain phosphodiesterases with GGDEF domains, the identity of archaeal cyclic di‐GMP receptors, and the physiological and metabolic role of cyclic di‐GMP signaling. On the phylogenetic level, it is of utmost impact to assess the origin of cyclic di‐GMP turnover domain proteins. Is there a common origin from LUCA, before, or are the cyclic di‐GMP turnover proteins entirely or to a large extent acquired by horizontal gene transfer from Bacteria? Another follow‐up question is whether cyclic di‐GMP turnover domains have evolved differently and/or faster than in Bacteria. Systematic analysis of domain composition between archaeal and bacterial proteins will provide indications for functional differentiation in response to signals distinct for specific ecological niches. As initial observation already indicated archaeal GGDEF domains uniquely deviating in the consensus catalytic GGDEF motif, the function of GGDEF domains which deviate substantially in the signature amino acids needs to be defined. Most importantly, the phylogenetic context of cyclic di‐GMP turnover proteins is an open question. Are GGDEF domain proteins present in all archaeal clades? So far, we have mainly investigated proteins from the DAPNN and II clade (with the archaeal origin of MBN1216112.1 still uncertain). Last but not least, as phenotypes are observed in the *S. typhimurium* model system, archaeal GGDEF domain protein can be used as a tool to identify novel regulatory pathways for motility, biofilm formation, and other physiological and metabolic traits in Gram‐negative and Gram‐positive Bacteria. In conclusion, the discovery of cyclic di‐GMP synthesizing GGDEF domain proteins present in Archaea opens questions of their phylogenetic origin, overall occurrence, function, and role in physiology, metabolism, and behavior in Archaea.

## 4 Experimental Procedures

### 4.1 Strains, Plasmid, and Growth Conditions

The archaeal isolate *M. arvoryzae* MRE50 (DSM 22066) (Sakai et al. 2010) was grown in DSM medium 1318 under anaerobic conditions at 45°C for 7 days. *H. salinisoli* KLK7 (KCTC 4307T; (Dong et al. 2024)) obtained from the Korean Collection for Type Cultures (KCTC) has been grown in KCTC medium No. 1762 HCM (Haloarchaeal complex medium) or on respective agar plates at 37°C. *H. salinisoli* KLK7 was stored in KCTC medium No. 1762 HCM with 50% glycerol at −80°C.

*E. coli* TOP10 has been used for most cloning events. *E. coli* TOP10 and derivatives were conventionally grown on LB agar plates or in LB medium at 37°C. *S. typhimurium* UMR1 (ATCC 14028 Nal^r^ rdar_28°C_) and *S. typhimurium* UMR1 Δ*yhjH* were maintained on LB medium plates and assessed on Congo red agar plates (LB medium without NaCl, 40 μg mL^−1^ Congo red, 20 μg mL^−1^ Coomassie Brilliant Blue G‐250). Antibiotics were added at the following concentration: Amp (100 μg mL^−1^) and Gm (30 μg mL^−1^). Induction of protein production was optimized using variable concentrations of IPTG from 0 to 0.75 mM and of L‐arabinose from 0% to 0.1%.

### 4.2 Cloning of Open Reading Frames

Synthetic gene templates of original and codon optimized open reading frames including the native or a mutated Shine‐Dalgarno sequence were amplified using specific or universal primers. Two different cloning strategies were applied in order to enable protein production using the pBAD28 vector under the control of the L‐arabinose inducible pBAD promoter. Initially, conventional cloning after restriction enzyme digest of the amplified gene fragment and the vector, subsequent in vitro ligation by T4 ligase and transformation into chemocompetent *E. coli* TOP10 (alternatively DH5α and XL‐1 Blue) cells was applied. In an alternative experimental approach, synthetic gene fragments were recombined by in vivo cloning via homologous vector sequences (Bubeck et al. 1993; Jones and Howard 1991). In detail, pBAD28 was amplified by PCR using Q5 polymerase and universal primers that included by default the XbaI and SphI restriction sites, respectively, to achieve a linear plasmid. Open reading frames were amplified using primers that contained the flanking pBAD28 nucleotide sequence at the 5′ end concluding with XbaI and SphI sites at the 3′ end, respectively. Both linear PCR products were transformed into chemocompetent *E. coli* TOP10 cells according to the standard protocol. Cells were subsequently plated onto LB medium containing the appropriate antibiotics to select for assembled plasmids.

The *adrA* open reading frame was recombined with plasmid pSRKGm under the control of the IPTG inducible *lac* promoter (Khan et al. 2008). The *adrA* open reading frame was amplified with primers that contained 15 bp homologous vector sequences with NdeI and NheI sites, respectively. pSRKGm was amplified by PCR using primers that included by default the NdeI and NheI sites that flank the multicloning site and the *lacZ*‐α fragment at the 5′ and the 3′ end of the vector sequences, respectively. The integrity of all cloned genes was confirmed by Sanger sequencing.

### 4.3 Site Directed Mutagenesis and Construction of Gene Deletions

Amino acid substitutions in order to abolish the catalytic activity of GGDEF and HD‐GYP domain proteins were introduced with the Q5 Quick change kit (New England Biolabs). Alternatively, the equivalent reaction using the individual components, Phusion polymerase to amplify the plasmid template with mutagenizing primers combined subsequently with parallel enzymatic reactions of polynucleotide kinase, T4 ligase, and DpnI was performed. In a conceptually different experimental approach, site‐directed mutagenesis has been performed using mutagenizing primers overlapping by at least 15 nucleotides to amplify the gene fragment containing vector. Circular nicked and the linear construct were transformed into chemocompetent *E. coli* K‐12 cells to be recombined in vivo.

Deletion of a fragment from a cloned gene was constructed by amplifying the vector containing the wild type gene with primers that define the deletion and overlap by 15 bps. The linearized vector was subsequently transformed into chemocompetent *E. coli* TOP10 cells for in vivo recombination cloning and cells with assembled vectors were selected on LB plates containing appropriate antibiotics. All constructs were confirmed by Sanger sequencing.

### 4.4 Plasmid Transformation

Plasmid constructs were transformed into chemocompetent *E. coli* TOP10 and into *S. typhimurium* UMR1 and derivatives by electroporation as described (Römling, Sierralta, et al. 1998).

### 4.5 Analysis of Gene Expression by qRT‐PCR

*M. arvoryzae* MRE50 and *H. salinisoli* KLK7 were grown in liquid culture in their respective media. Subsequently, hot‐acidic‐phenol extraction was used to purify total RNA (Römling, Bian, et al. 1998). Cells were lysed in a buffer containing 1% sodium dodecylsulfate, protein and DNA were repeatedly extracted by hot‐acidic‐phenol, while residual DNA was subsequently removed by DNase I treatment. RNA concentration, quality, and residual contamination were assessed by estimation of the 260/280 nm ratio from the 210 to 400 nm spectrum, agarose gel electrophoresis, and by control PCR with the target gene primers for the housekeeping gene *rplJ*. RNA was reversely transcribed with the High‐Capacity cDNA Reverse Transcription Kit (Applied Biosystems). Quantitative PCR was performed monitored by SYBR Green incorporation (PCR Master mix by Applied Biosystems) in a Rotor‐Gene PCR cycler (Corbett Life Science). The arithmetic mean of three experiments and the standard deviation were calculated in Excel. Data were normalized to the expression level of the *rplJ* reference gene.

### 4.6 Biofilm Formation Assay

Isolates were streaked for single colonies or 10 μL of a 5 OD_600_ suspension was spotted on Congo red agar plates. Congo red agar plates were incubated at 28°C (alternatively at 37°C and 42°C) for up to 72 h with positive control: *S. typhimurium* UMR1 and/or UMR1 Δ*yhjH* and negative control: *S. typhimurium* UMR1 Δ*csgBA* Δ*bcsA* containing the respective empty vector. Colony morphology and coloration were documented in a gel documentation chamber using a conventional mobile phone.

### 4.7 Motility Assay

Two μl of a bacterial suspension of 5 OD_600_ were inoculated into a motility agar plate (LB medium with 0.25% agar). The plate was incubated at 37°C for up to 24 h. The zone of motility was documented in a gel documentation chamber (Biorad) using a conventional mobile phone and subsequently the diameter of the motility zone was calculated. Positive control was *S. typhimurium* UMR1 and negative control *S. typhimurium* MAE108 (UMR1 Δ*fliC* Δ*fljB*) containing the respective empty vector.

### 4.8 Assessment of Relative Cyclic di‐GMP Concentrations by the Vc2*lacZY* Riboswitch Assay

The Vc2 aptamer was used as a cyclic di‐GMP sensor to qualitatively assess cyclic di‐GMP synthesis and degradation in the context of the riboswitch which subsequently regulates production of the downstream β‐galactosidase LacZY protein. The cyclic di‐GMP sensing unit integrated in single copy into the Tn*7* site of *E. coli* TOP10 inhibits production of the β‐galactosidase in response to high cyclic di‐GMP concentrations due to conformational alterations upon binding. Activation of protein production in response to low cyclic di‐GMP upon degradation by phosphodiesterases is most sensitively monitored in the background of elevated cyclic di‐GMP levels (concomitant expression of a diguanylate cyclase; (Liu et al. 2018, 2020)). 5‐bromo‐4‐chloro‐3‐indolyl‐β‐D‐galactopyranoside (X‐gal; 80 μg mL^−1^) was the substrate to monitor β‐galactosidase activity.

Vectors containing genes and variants cloned into pBAD28 were transformed into chemocompetent *E. coli* TOP10 Tn*7*::Vc2*lacZY* to assess diguanylate cyclase activity or into chemocompetent *E. coli* TOP10 Tn*7*::Vc2*lacZY* pSRKGm‐AdrA to assess phosphodiesterase activity. Three μl of a logarithmically grown suspension of OD_600_ = 1 were spotted onto a LB agar plate containing 0% and 0.1% L‐arabinose and appropriate antibiotic(s) with the degree of β‐galactosidase activity assessed after 24, 48, and 72 h.

AdrA of *S. typhimurium* UMR1 and its catalytically incompetent variant (Römling et al. 2000; Simm et al. 2004), CdaS of *Bacillus subtilis* 3A1 and its catalytically incompetent variant (Mehne et al. 2014), and DncV of *E. coli* ECOR31 and its catalytically incompetent variant (Li et al. 2019) cloned in pBAD28 served as cyclic di‐nucleotide synthesis and specificity controls. YhjH and its catalytic mutants E48A and E136A cloned in pBAD28 (Simm et al. 2004) served as phosphodiesterase activity controls.

### 4.9 Bioinformatic Analysis—Identification and Verification of GGDEF Domain Proteins

Initially, the GGDEF domain of AdrA (YaiC) of *S. typhimurium* ATCC 14028 (Römling et al. 2000) was used as a query searching the Archaea (ID: 2157) phylogenetic domain of non‐redundant sequences with BLAST (Altschul et al. 1990) to retrieve candidate proteins containing GGDEF domains. Of note, we previously observed that searching with the GGDEF domain of AdrA retrieves the majority of available GGDEF domains (Liu et al. 2020). Subsequently, results were screened for GGDEF domain proteins encoded by genomes of archaeal isolates rather than identified in metagenome‐assembled genomes (MAGs). As a second step, GGDEF domain proteins encoded by MAGs were verified as derived from archaeal sequences by assessment of the phylogenetic origin of at least two proteins encoded in close vicinity up‐ and downstream of the GGDEF domain protein gene, respectively. This assessment served as a proxy for the phylogenetic origin of the respective contig. Sufficiently long contigs to reliably assess the archaeal origin of the GGDEF genes were preferred.

### 4.10 Bioinformatic Analysis—Other Analytical Approaches

GGDEF domain proteins were used as query searching the Archaea (ID: 2157) and Bacteria (ID: 2) phylogenetic domains with BLAST to obtain homologs (Altschul et al. 1990). Protein domains were identified during the BLAST search which provides cumulative results from the Clusters of Orthologous Genes (COG), Conserved Domain Database, Protein Family Models (NF), InterPro/Pfam, TIGR, SMART databases. Predictions of alternative open reading frames were assessed with ORF Finder at the NCBI website (ncbi.nlm.nih.gov/orffinder/). Transmembrane helices were evaluated with TMHMM (Moller et al. 2001). Identity and similarity of the proteins were calculated with the Sequence Manipulation Suite (Stothard 2000) after sequence alignment by ClustalX 2.1 and, if required, manual curation. Domain structures were displayed with Illustrator for Biological Sequences IBS2.0 (Xie et al. 2022). Structural models were retrieved from the UniProt database (AlphaFold; (UniProt 2025)) and/or calculated using Phyre2 (Kelley et al. 2015) and AlphaFold 3 (Jumper et al. 2021) to identify additional domain folds. Structural homologs were identified by screening databases with DALI (Holm et al. 2023) and FoldSeek (van Kempen et al. 2024). Structural models were displayed and compared (MatchMaker function) in Chimera 1.18 (Pettersen et al. 2004). Homologous GGDEF open reading frames in the context of their gene neighborhood were displayed with Easyfig (Sullivan et al. 2011) using annotated gene bank files comprising +/− 10,000 bps up‐ and downstream of the respective GGDEF gene. Most similar GGDEF domains to archaeal and all GGDEF domains were identified by the BLAST search restricting the results to 500 for all non‐redundant proteins. Multisequence alignment was performed with ClustalX 2.1 (Higgins and Sharp 1988) and manually curated using GeneDoc (Nicholas et al. 1997) to obtain alignments of only the homologous GGDEF domain sequences common to all proteins. If enhanced visibility of phylogenetic trees was required, proteins and/or domains with > 90% or > 70% similarity were removed using Jalview (Waterhouse et al. 2009). Alignments were displayed with ESPript 3.0 (Robert and Gouet 2014). Neighborhood joining (to test feasibility) and maximum likelihood‐based phylogenetic trees using 1000 (occasionally 100) bootstraps were calculated and displayed in MEGA 7.0 or MEGA 11.0 (Kumar et al. 2016) using standard parameters.

## Author Contributions

**Roman Sidorov:** investigation, data curation, formal analysis, writing – review and editing. **Li Li:** data curation, formal analysis, investigation, methodology, writing – review and editing. **Ali Dadvar:** investigation, formal analysis, data curation, writing – review and editing. **Irfan Ahmad:** data curation, investigation, formal analysis, writing – review and editing. **Stefan Spring:** investigation, methodology, writing – review and editing. **Jörg Overmann:** investigation, data curation, writing – review and editing. **Ute Römling:** conceptualization, methodology, data curation, investigation, validation, formal analysis, supervision, resources, project administration, funding acquisition, visualization, writing – original draft, writing – review and editing.

## Funding

This work has been supported by a grant from the Swedish Research Council for Natural and Engineering Sciences (diary number 2022‐04865) to Ute Römling and the Karolinska Institutet (KI Funds 2023).

## Supporting information


**Figure S1:** Characterisation of archaeal isolates.
**Figure S2:** Genomic localization of the GGDEF domain proteins from isolates.
**Figure S3:** Overlay of the Alphafold 3 structural model of the REC_S_ domain of GGDEF_MRE50 (WP_012035199.1; cyan) with the REC receiver domain (PDB: 7W9H) crystal structure of the δ^54^ä‐dependent transcription factor FleR (grey).
**Figure S4:** Structural and phylogenetic analysis of GGDEF domains and GGDEF domain proteins GGDEF_MRE50 (WP_012035199.1) from *M. arvoryzae* MRE50, GGDEF_KLK7 (WP_339106355.1) from *H. salinisoli* KLK7 and GGDEF_E3 (WP_363463662.1) from *H. borinquense* E3.
**Figure S5:** AlphaFold 3 structural model of dimer formation of the wild type full length protein GGDEF_MRE50 (WP_012035199.1) from *M. arvoryzae* MRE50.
**Figure S6:** Phylogenetic tree of Archaea with phylogenetic position of 16 additional GGDEF domain proteins identified in archaeal MAGs.
**Figure S7:** Alignment of 21 GGDEF domains of candidate archaeal proteins from MAGs with reference GGDEF domains and class I, II, and III GGDEF domains.
**Figure S8:** Assessment of catalytic activity of archaeal GGDEF domain proteins from MAGs.
**Figure S9:** In vivo assays to assess the catalytic activity of GGDEF domain proteins UCD02967.1, HJJ27798.1, and RJQ22058.1 from metagenome‐assembled genomes.
**Table S1:** Strains used in this study.
**Table S2:** Plasmids used in this study.
**Table S3:** Primers used in this study.

## Data Availability

The data that supports the findings of this study are available in the Supporting Information of this article and/or available upon request from the authors.
