# Supplementary material for: Archaeal Genes Code for GGDEF Domain Proteins With Diguanylate Cyclase Activity
Source: Mol Microbiol. 2026 Sep 21;126(5):576–95. doi: 10.1111/mmi.70117 (PMC13725321; doi:10.1111/mmi.70117)
Supplement: Supplementary file 1 [file MMI-126-576-s001.docx]

**Supporting Information**

**Archaeal genes code for GGDEF domain proteins with diguanylate cyclase activity**

Roman Sidorov^1,*^, Li Li^1,*^, Ali Dadvar^1,*^, Irfan Ahmad^1,&^, Stefan Spring^2^, Jörg Overmann^2,3,#^, Ute Römling^1^

^1^Department of Microbiology, Tumor and Cell Biology, Biomedicum, Karolinska Instittuet, Stockholm, Sweden

^2^Leibniz Institute DSMZ-German Collection of Microorganisms and Cell Cultures, Inhoffenstraße 7B, 38124 Braunschweig, Germany

^3^Braunschweig University of Technology, Spielmannstraße 7, 38106 Braunschweig, Germany

*contributed equally

^&^current address: Department of Cell and Molecular Biology, SciLifeLab, Uppsala University, Sweden

^#^current address: Bavarian State Collections of Natural History; Munich, Germany; and Chair for Molecular Diversity Research, Faculty of Biology, Ludwig-Maximilians-University Munich; Munich, Germany

Corresponding author:

Ute Römling, ute.romling@ki.se

**Content	page**

Supporting Figure 1	3

Supporting Figure 2	5

Supporting Figure 3	7

Supporting Figure 4	8

Supporting Figure 5	13

Supporting Figure 6	14

Supporting Figure 7	15

Supporting Figure 8	17

Supporting Figure 9	19

References	20

Supporting data, Materials and Experimental Procedures	21

**Additional supporting information**

Supporting TABLE S1	38

Supporting TABLE S2	39

Supporting TABLE S3	43

**Supporting Figures**

**Supporting Figure 1** Characterisation of archaeal isolates.

**Figure S1A**. Phylogenetic tree of Halobacteria with the position of GGDEF domain proteins GGDEF_KLK7 (WP_339106355.1) from *Haloterrigena salinisoli* KLK7 and GGDEF_E3 (WP_363463662.1) from *Halogeometricum borinquense* E3. Phylogenetic tree of Halobacteria has been redrawn from (Blanquart, Groussin et al., 2021).

**Figure S1B** Micrographs and plate-grown archaeal isolates. Left, *M. arvoryzae* MRE50 grown in DSM medium 1318 under anaerobic conditions at 45°C for seven days. Middle, H. *salinisoli* KLK7 grown in KCTC medium No. 1762 HCM. Right, *H. salinisoli* KLK7 grown on KCTC medium No. 1762 HCM medium for 14 days as 37 °C.

**Supporting Figure 2** Genomic localization of the GGDEF domain proteins from isolates.

**Figure S2A.** Chromosomal location of the Pac-PAS/PAC-PocR-GGDEF-HD-GYP domain protein genes from *Methanocella* spp. Three genomes of isolates belonging to the *Methanocella* genus encode the Rec-PAS/PAC-PocR-GGDEF-HD-GYP domain protein gene. In all three isolates the Rec-PAS/PAC-PocR-GGDEF-HD-GYP domain protein gene *RCIX2274* is present at the same chromosomal location flanked by HEAT repeat domain-containing protein genes *RCIX2272* and *RCIX2275*. Of note, the three *Methanocella* isolates are of distinct geographical origin. While *M. arvoryzae* MRE50 has been isolated at the Italian Rice Research Institute, Vercelli, Italy, *Methanocella* sp. MCL-LM from a biocrust at Midreshet Ben-Gurion, Israel and *Methanocella* sp. SMAG_U12379 from a soil metagenome, Colombia. BLAST-based gray scale indicates percent sequence identity.

**Figure S2B.** Genome location of the GGDEF-unknown domain protein genes from *H. salinisoli* KLK7 and *H. borinquense* E3. The genome location of the two proteins is distinct. WD430_RS22260 encoding GGDEF_KLK7 (WP_339106355.1) is located on the plasmid pu3 of *H. salinisoli* KLK7, while R2Z48_RS01700 encoding GGDEF_E3 (WP_363463662.1) of *H. borinquense* E3 is located on the chromosome. BLAST-based gray scale indicates percent sequence identity.

**Supporting Figure 3** Overlay of the Alphafold 3 structural model of the REC_S_ domain of GGDEF_MRE50 (WP_012035199.1; cyan) with the REC receiver domain (PDB: 7W9H) crystal structure of the δ^54^ä-dependent transcription factor FleR (grey).

Left, The configuration at the phosphorylgroup acceptor site of the receiver domain. The D/E_9_D_10_D_53_K_103_ motif of the FleR receiver domain is indicated in green D_53_ receives the phosphorylgroup. D_14_ and D_58_ as potential receivers of a phosphorylgroup in GGDEF_MRE50 are indicated in red. Right, Structural similarity versus non-canonical amino acid sequences in the REC_S_ receiver domain of GGDEF_MRE50 compared to FleR. Red arrow, first β-strand of the receiver domain of FleR at lysine K_4_ colocalizes with β3 of GGDEF_MRE50 at aspartate D_51_. Blue arrow, First β-strand of the receiver domain of GGDEF_MRE50 at leucine L_9_ colocalizes with β3 of FleR at leucine L_49_ .

**Supporting Figure 4** Structural and phylogenetic analysis of GGDEF domains and GGDEF domain proteins GGDEF_MRE50 (WP_012035199.1) from *M. arvoryzae* MRE50, GGDEF_KLK7 (WP_339106355.1) from *H. salinisoli* KLK7 and GGDEF_E3 (WP_363463662.1) from *H. borinquense* E3.

**Figure S4A.** Structural models and domain structures of bacterial proteins MGB9858445.1 and MBS3969924.1 which possess a GGDEF domain most similar to GGDEF_MRE40 (WP_012035199.1) from *M. arvoryzae* MRE50. Structural models were created with AlphaFold 3 (Jumper, Evans et al., 2021) and processed in Chimera 1.18 (Pettersen, Goddard et al., 2004). Domain structure of proteins were constructed in IBS 2.0 (Xie, Li et al., 2022).

**Figure S4B.** Phylogenetic tree of archaeal GGDEF domain proteins with the GGDEF domains most similar to the GGDEF domain of GGDEF_MRE50 (WP_012035199.1) from *M. arvoryzae* MRE50. Proteins were retrieved after BLAST search using the GGDEF domain of GGDEF_MRE50 as a query. After alignment with ClustalX2 and manual curation in GeneDoc, phylogenetic assessment was performed in MEGA 7.0 using the Maximum Likelihood approach and statistical significance of the tree was assessed with 100 bootstraps. Of 500 domains, domains with <70% identity are displayed.

WebLogo 3 (Crooks, Hon et al., 2004) of 500 most similar archaeal GGDEF domains with signature amino acids required for catalysis indicated. Stars indicate I-site contributing arginines.

**Figure S4C.** Phylogenetic tree of all GGDEF domain proteins with the GGDEF domains most similar to the GGDEF domain of GGDEF_MRE50 (WP_012035199.1) from *M. arvoryzae* MRE50. Proteins were retrieved after BLAST search using the GGDEF domain of GGDEF_MRE50 as a query. Of 500 domains, domains with >90% identity are displayed.

WebLogo 3 (Crooks et al., 2004) of 500 most similar GGDEF domains with signature amino acids required for catalysis indicated. Stars indicate I-site contributing arginines.

**Figure S4D.** Phylogenetic tree of archaeal GGDEF domain proteins with the GGDEF domains most similar to the GGDEF domain of GGDEF_KLK7 (WP_339106355.1) from *H. salinisoli* KLK7. Proteins were retrieved after BLAST search using the GGDEF domain of GGDEF_KLK7 as a query. Of 500 domains, domains with <90% identity are displayed.

WebLogo 3 (Crooks et al., 2004) of 500 most similar archaeal GGDEF domains with signature amino acids required for catalysis indicated. Stars indicate I-site contributing arginines.

**Figure S4E.** Phylogenetic tree of all GGDEF domain proteins with the GGDEF domains most similar to the GGDEF domain of GGDEF_KLK7 (WP_339106355.1) from *H. salinisoli* KLK7 with domains <90% identity displayed. Proteins were retrieved after BLAST search using the GGDEF domain of GGDEF_KLK7 as a query. WebLogo 3 (Crooks et al., 2004) of 500 most similar GGDEF domains with signature amino acids required for catalysis indicated. Stars indicate I-site contributing arginines.

**Supporting Figure 5** AlphaFold 3 structural model of dimer formation of the wild type full length protein GGDEF_MRE50 (WP_012035199.1) from *M. arvoryzae* MRE50.

The monomers and their GGDEF domains (in green) are arranged oppositely contrasting a catalytic competent arrangement. Blue, HD-GYP domain; red, linker.

**Supporting Figure 6** Phylogenetic tree of Archaea with phylogenetic position of 16 additional GGDEF domain proteins identified in archaeal MAGs.

Red circles, proteins from archaeal isolates investigated, but not named here; green circles, proteins experimentally investigated; blue circles, proteins verified to be encoded by archaea; red triangle and dark red text, Diapherotrites encoded protein to be investigated further. MBN121612.1, archaeal origin not confirmed. Phylogenetic tree has been redrawn from (Aouad, Flandrois et al., 2022).

**Supporting Figure 7** Alignment of 21 GGDEF domains of candidate archaeal proteins from MAGs with catalytically active reference GGDEF domains and class I, II and III GGDEF domains.

GGDEF domains from well investigated reference proteins are PleD from *Caulobacter vibrioides* CB15 (CC_2462, PleD_CAUCRE) and WspR from *Pseudomonas aeruginosa* PAO1 (PA3702, WspR_PSEAI). GGDEF domain starts with the conserved aspartate at arbitrary position 9. Conserved signature motifs are indicated: light plum, wide turn; red, substrate interacting residues; green, Mg^2+^ binding; light blue, stabilizing the transition state; blue, allosteric I-site; violet, probable dimer interface; yellow, GG[D/E]EF motif; underlined, salt bridge. Light blue side bar, reference GGDEF domains; violet bar, GGDEF domains from archaea; green side bar, catalytically active GGDEF domains; yellow bar, domains with unconventional signature motifs; red, catalytically inactive GGDEF domains. Protein designation in Supporting Materials and Experimental Procedures. Alignment was created with ClustalX 2.1 (Higgins and Sharp 1988), manually curated in GeneDoc and visualized with ESPript 3.0 (Robert and Gouet 2014). Secondary structure of PleD from 2V0N (RCSB Protein Data Bank, RCSB PDB). *acc*, accessibility. Relative accessibility in blue >0.4 to 1, cyan and white <0.1 burried indicated in the bottom bar.

**Supporting Figure 8** Assessment of catalytic activity of archaeal GGDEF domain proteins from MAGs.

**Figure S8A** *In vivo* Vc2 riboswitch assay in *E. coli* TOP10 Tn*7*::Vc2*lacZY* to assess catalytic activity of the controls for the diguanylate cyclase (DGC) and cyclic di-GMP specific phosphodiesterase (PDE) assay. Cyclic di-nucleotide controls for cyclic di-GMP were *E. coli* TOP10 Tn*7*::Vc2*lacZY* pAdrA and the pAdrA G288A, G289A mutant; pDncV and its catalytic pDncV D129A, D131A mutant and pCdaS and its catalytic pCdaS D135A mutant. The PDE assay expressed pSRKGm-AdrA per default without IPTG induction with PDE activity to be observed by relieve of repression of translation. WT, wild type protein. VC is pBAD28. All genes cloned in pBAD28.

**Figure S8B** Quantitative estimation of flagella based swimming motility to assess the catalytic activity of MAGs derived GGDEF domain proteins and its variants in *S. typhimurium* UMR1 Δ*yhjH*. p*<0.05, p**<0.01, p***<0.001 by student’s t-test compared to *S. typhimurium* UMR1 Δ*yhH* vector control pBAD28 and negative control *S. typhimurium* MAE108 (Δ*fliC* Δ*fljB*) pBAD28.

**Supporting Figure 9** *In vivo* assays to assess the catalytic activity of GGDEF domain proteins UCD02967.1, HJJ27798.1 and RJQ22058.1 from metagenome-assembled genomes.

Rdar biofilm formation and flagella based swimming motility in the *S. typhimurium* UMR1 and UMR1 Δ*yhjH* model and Vc2 riboswitch assay in *E. coli* TOP10 Tn*7*::Vc2*lacZY* to assess the catalytic activity of GGDEF domain proteins UCD02967.1, HJJ27798.1 and RJQ22058.1 from metagenome-assembled genomes and its catalytic variants. Assay conditions as described in Figure 3A. Genes and variants have been cloned into the pBAD28 vector. Induction of protein production with 0.1% L-arabinose.

**References**

Aouad, M., Flandrois, J.P., Jauffrit, F., Gouy, M., Gribaldo, S. & Brochier-Armanet, C. (2022) A divide-and-conquer phylogenomic approach based on character supermatrices resolves early steps in the evolution of the archaea. *BMC Ecol Evol* **22**: 1.

Blanquart, S., Groussin, M., Le Roy, A., Szollosi, G.J., Girard, E., Franzetti, B. et al. (2021) Resurrection of ancestral malate dehydrogenases reveals the evolutionary history of halobacterial proteins: Deciphering gene trajectories and changes in biochemical properties. *Mol Biol Evol* **38**: 3754-3774.

Crooks, G.E., Hon, G., Chandonia, J.M. & Brenner, S.E. (2004) Weblogo: A sequence logo generator. *Genome Res* **14**: 1188-1190.

Dong, X.Y., Mao, Y.L., Zhang, Q.K., Zhu, L.R., Hou, J. & Cui, H.L. (2024) Genome‑based classification of the family Natrialbaceae and description of four novel halophilic archaea from three saline lakes and a saline-alkaline land. *Extremophiles* **28**: 47.

Guzman, L.M., Belin, D., Carson, M.J. & Beckwith, J. (1995) Tight regulation, modulation, and high-level expression by vectors containing the arabinose pBAD promoter. *J Bacteriol* **177**: 4121-4130.

Higgins, D.G. & Sharp, P.M. (1988) Clustal: A package for performing multiple sequence alignment on a microcomputer. *Gene* **73**: 237-244.

Jumper, J., Evans, R., Pritzel, A., Green, T., Figurnov, M., Ronneberger, O. et al. (2021) Highly accurate protein structure prediction with Alphafold. *Nature* **596**: 583-589.

Khan, S.R., Gaines, J., Roop, R.M., 2nd & Farrand, S.K. (2008) Broad-host-range expression vectors with tightly regulated promoters and their use to examine the influence of TraR and TraM expression on Ti plasmid quorum sensing. *Appl Environ Microbiol* **74**: 5053-5062.

Li, F., Cimdins, A., Rohde, M., Jänsch, L., Kaever, V., Nimtz, M. et al. (2019) DncV synthesizes cyclic GMP-AMP and regulates biofilm formation and motility in *Escherichia coli* ECOR31. *mBio* **10**:

Liu, Y., Kim, H. & Römling, U. (2018) In vivo analysis of cyclic di-GMP cyclase and phosphodiesterase activity in *Escherichia coli* using a Vc2 riboswitch-based assay. *Bio Protoc* **8**: e2753.

Pettersen, E.F., Goddard, T.D., Huang, C.C., Couch, G.S., Greenblatt, D.M., Meng, E.C. et al. (2004) Ucsf chimera--a visualization system for exploratory research and analysis. *J Comput Chem* **25**: 1605-1612.

Robert, X. & Gouet, P. (2014) Deciphering key features in protein structures with the new endscript server. *Nucleic Acids Res* **42**: W320-324.

Römling, U., Bian, Z., Hammar, M., Sierralta, W.D. & Normark, S. (1998) Curli fibers are highly conserved between *Salmonella typhimurium* and *Escherichia coli* with respect to operon structure and regulation. *J Bacteriol* **180**: 722-731.

Sakai, S., Conrad, R., Liesack, W. & Imachi, H. (2010) *Methanocella arvoryzae* sp. Nov., a hydrogenotrophic methanogen isolated from rice field soil. *Int J Syst Evol Microbiol* **60**: 2918-2923.

Simm, R., Lusch, A., Kader, A., Andersson, M. & Römling, U. (2007) Role of eal-containing proteins in multicellular behavior of *Salmonella enterica* serovar Typhimurium. *J Bacteriol* **189**: 3613-3623.

Simm, R., Morr, M., Kader, A., Nimtz, M. & Römling, U. (2004) GGDEF and EAL domains inversely regulate cyclic di-GMP levels and transition from sessility to motility. *Mol Microbiol* **53**: 1123-1134.

Xie, Y., Li, H., Luo, X., Li, H., Gao, Q., Zhang, L. et al. (2022) Ibs 2.0: An upgraded illustrator for the visualization of biological sequences. *Nucleic Acids Res* **50**: W420-W426.

**Supporting data**

**Materials and Experimental Procedures**

**Gene templates used in this study**

**>WP_012035199.1 (GGDEF_MRE50) Q0W2L1_METAR Signal transduction protein OS=*Methanocella arvoryzae* (strain DSM 22066 / NBRC 105507 / MRE50) OX=351160 GN=RCIX2274 PE=4 SV=1**

**LRTGEDSV**LAGINGDVLLLTGDGAEVAAIGEALALAGLDYRHAVDVASACFLAADTAFDAVLLGSDQSVSAAIAFLKNSGTQVPVIVMSGTERCDMPVEAIKYVLSGALVQVAGLQCPGSGAIAAILETISRNRTRNLGARLQDEVNKLSYIADTFPECLLMIDRAGTIVYANRACYETYEYSNGALKSCNISGLLAGEADFTKLKELLRVTRSGGSNTELATISRRGQKRLSLAVITPRSDDAGAIFAYVILLQDITDSRYAETRMERLISALTSPEAGLSSLDFRDLITDPEIQVMQDTLAAAMGVSAVIVTPGGTPLNQRSNVTSLCSSLARPGTVSAQICGKCITDLAGRADAGQQARCTCPCTGMTEVAIPLVVNGKPAAFWIVGQVSLGEPAREKLQDLLTDQGISPADVRNLIAGVHRMTEHQLNQIVATFSMVADKVTRLAIQNFRQGKYINERNTTLEELQRSERRLQEMSYRDGLTGLHNRAYFEQELGRLESASSAFPVSIVAADVDGLKLINDTLGHTAGDQHLKVAGNMISAVFSASGDVCRTGGDEFCILLPGCGEAAAGKMLELLREEIRRYNERQPGPPLSISTGIAAINPGESLYDAYKRADRLMYEHKLKRDDYARSKSIDMILLSLSERDYMDHGHTRVLTVIVDAMASELGLGEDARANLKLLARLHDLGKIGVPDEIVMKPAKLDEHEWVLMRSHVEIGANIASRSLHMNHIAPLIRHHHERYDGTGYPSGLSGSQIPLECRIMAIADSYDAMIADRPYRKGLSHECALEEILRNAGTQFDPEIVKVFAGIVRSGRL

**Synthax for all gene fragments**

Underlined are start and stop codons; in italics codons for 6xHis-tag, in red, regions complementary to the pBAD30/28 vector and in bold mutated nucleotides. Bold and underlined is the sequence present in each of two synthetic gene fragments. For in vivo cloning mimicking conventional restriction enzyme cloning, the gene fragment is flanked by the XbaI and SphI sites (red and underlined) located within the multicloning site of pBAD30/28 vector.

**Codon optimized gene template for GGDEF_MRE50 (WP_012035199.1) from** *Methanocella arvoryzae* MRE50

GTACCCGGGGATCCTCTAGAGATAGT**T**G**A**CGAAA**T**GCGGGCTGAGAACAGGCGAGGATTCAGTTTTGGCCGGAATCAATGGTGACGTCTTACTTCTTACGGGAGACGGTGCTGAGGTTGCTGCGATTGGAGAGGCCCTTGCGTTAGCCGGGCTGGATTACCGTCACGCCGTCGATGTGGCTTCAGCCTGCTTTTTAGCCGCGGACACGGCCTTTGACGCTGTACTGCTGGGATCAGATCAATCTGTGAGTGCAGCGATCGCCTTTCTTAAAAATTCCGGGACGCAGGTACCGGTTATCGTGATGTCAGGGACCGAGCGTTGCGACATGCCAGTAGAAGCAATTAAATATGTATTGAGCGGAGCTCTTGTACAGGTCGCCGGATTACAGTGCCCCGGTTCTGGTGCGATTGCGGCCATCTTGGAAACGATCTCACGCAACCGCACTCGCAATCTGGGTGCGCGTCTGCAGGACGAGGTTAACAAGCTGAGTTATATCGCAGATACGTTTCCAGAATGCTTGTTAATGATCGATCGCGCAGGTACCATCGTGTATGCCAATCGCGCTTGCTACGAGACGTATGAGTACTCGAATGGCGCACTTAAATCTTGTAATATCTCTGGACTTTTGGCCGGGGAAGCGGATTTTACAAAGCTGAAAGAATTATTGCGCGTGACACGCTCGGGCGGATCCAATACCGAGTTGGCCACGATTAGTCGTCGTGGGCAGAAACGTCTGTCTCTGGCGGTTATCACGCCACGCAGTGACGATGCTGGTGCAATCTTTGCATATGTAATCTTGTTACAGGACATCACGGATTCACGCTATGCGGAAACCCGCATGGAGCGCCTTATCAGTGCTCTGACCTCGCCCGAGGCTGGGCTGTCGTCGCTTGATTTTCGCGATCTTATTACGGACCCGGAAATCCAGGTTATGCAAGATACTCTTGCCGCAGCAATGGGGGTCTCGGCGGTAATTGTCACTCCTGGCGGCACGCCGCTGAATCAACGTTCTAACGTGACGTCTTTATGTTCCTCTCTTGCCCGCCCGGGGACTGTTAGCGCCCAAATTTGTGGTAAATGTATCACAGATTTAGCGGGGCGTGCAGACGCGGGACAGCAAGCTCGCTGCACGTGCCCGTGCACCGGTATGACGGAAGTGGCTATCCCCTTAGTGGTTAACGGCAAGCCCGCGGCATTCTGGATTGTAGGCCAGGTTTCGTTGGGTGAACCAGCTCGTGAAAAGCTTCAGGACCTGCTTACCGATCAAGGAA**TTTCCCCGGCGGACGTACGC**AACCTTATTGCCGGGGTGCATCGCATGACCGAACACCAATTAAATCAAATTGTTGCCACTTTTTCCATGGTAGCTGACAAAGTCACCCGCCTGGCGATTCAAAATTTTCGCCAAGGGAAGTATATCAACGAGCGTAACACGACGCTTGAAGAGTTGCAGCGCTCGGAACGCCGTTTGCAGGAAATGTCCTACCGCGATGGCTTGACAGGATTGCACAACCGTGCCTACTTCGAACAGGAATTAGGGCGTCTTGAGAGTGCTTCGTCTGCCTTCCCTGTGTCTATCGTAGCAGCGGACGTTGATGGATTGAAACTTATCAACGACACATTGGGTCATACTGCGGGGGATCAGCACTTAAAAGTTGCTGGGAACATGATTTCTGCAGTGTTCTCTGCATCAGGGGACGTCTGCCGTACCGGAGGTGATGAATTTTGCATCCTTCTTCCCGGTTGCGGAGAGGCGGCTGCAGGGAAAATGTTGGAATTGTTACGCGAGGAGATCCGTCGCTATAATGAGCGTCAACCGGGCCCTCCATTATCTATTTCCACTGGAATTGCGGCAATTAATCCTGGGGAAAGCTTATACGACGCTTATAAACGCGCCGATCGTTTGATGTACGAGCATAAACTTAAACGCGACGACTACGCGCGTTCTAAGTCTATTGATATGATCTTATTAAGTTTGTCCGAACGCGATTATATGGATCACGGTCACACACGTGTGCTGACGGTGATTGTCGATGCGATGGCCTCCGAATTGGGATTAGGCGAAGACGCCCGCGCTAACTTAAAGTTGCTTGCTCGTTTGCACGACCTTGGGAAGATCGGGGTACCAGACGAGATCGTTATGAAGCCTGCTAAGCTTGACGAACACGAGTGGGTTCTTATGCGTAGTCATGTTGAGATCGGAGCGAATATTGCCAGTCGTTCATTACACATGAACCACATTGCACCCTTAATCCGCCACCATCACGAGCGTTATGATGGGACTGGGTATCCGTCAGGCCTGTCTGGAAGCCAGATTCCCCTTGAATGTCGTATCATGGCTATTGCCGATTCGTACGACGCTATGATTGCGGACCGTCCGTACCGCAAAGGCTTGTCTCATGAATGTGCGTTGGAAGAAATTCTTCGTAATGCGGGCACACAGTTCGATCCCGAAATTGTTAAAGTATTCGCCGGTATTGTTCGCTCTGGACGTCTG*CATCACCACCACCATCAT*TAAGCATGCAAGCTTGGCTGTTT

.

>**WP_339106355.1 (GGDEF_KLK7)** ***Haloterrigena salinisoli* strain KLK7 lcl|ORF19**

**LLSFSLYATKILSNLNDLFGIL**MVDEHNVVSYGYIDLDGFSEINNEHGHTFGDKIIEEVVKFGRRFSDSKWEFNREYGQGDEFLMVLPDEDKESAVEVMEEFLKKLRSLRPEGQVVTASIGVATLPEDGANEGEITEKADLAMLNVEKWGGDGVIAYGNEKPTKEVEVWFEEAMLVEQDDLVKIQKWIDDSPSIRALKIYNESKGIPNESRSSKTMMSTTKYEGEIKGIVKEIKEWDSSKVRFEMRGDKIQLEKAGF

**Codon optimized gene template for GGDEF_KLK7 (WP_339106355.1) from *Haloterrigena salinisoli* KLK7**

GTACCCGGGGATCCTCTAGACAAAGGATGAAATCAGCCTACTGTTGAGTTTTTCTCTATATGCAACCAAAATATTAAGTAATCTCAATGACTTGTTTGGCATACTCATGGTAGATGAACATAACGTTGTTTCTTACGGTTACATTGACTTAGACGGCTTCTCTGAGATCAATAACGAACACGGCCACACCTTCGGCGATAAAATAATAGAAGAAGTCGTTAAATTCGGTAGAAGGTTTTCGGATTCTAAATGGGAATTCAATCGGGAATACGGACAGGGTGACGAGTTTTTGATGGTCTTACCTGATGAAGATAAGGAATCAGCGGTAGAGGTGATGGAAGAGTTTCTTAAAAAGTTACGTTCGCTCCGACCGGAAGGTCAGGTTGTCACCGCCAGTATTGGTGTAGCTACGTTACCCGAAGATGGAGCAAACGAAGGTGAGATCACTGAAAAAGCAGACCTTGCGATGCTGAATGTCGAGAAATGGGGCGGAGACGGTGTAATTGCATATGGTAACGAAAAGCCAACGAAAGAAGTAGAGGTTTGGTTTGAAGAAGCCATGTTGGTAGAGCAGGATGATCTTGTTAAAATACAGAAATGGATTGATGATAGCCCCTCAATTAGAGCACTGAAAATATATAACGAATCCAAAGGCATCCCTAACGAAAGTAGATCGTCAAAGACGATGATGTCCACGACAAAATACGAGGGGGAAATAAAAGGGATAGTTAAAGAGATCAAGGAGTGGGATTCCTCAAAAGTAAGGTTTGAGATGAGAGGTGATAAGATACAATTAGAAAAAGCAGGGTTC*CATCACCATCACCATCAC*TAAGCATGCAAGCTTGGCTGTTT

.

**>WP_363463662.1 (GGDEF_E3) GGDEF domain-containing protein *[Halogeometricum borinquense*]**

**LCEGVRKTPVHKQFY**LGISNLAKILSNPDGLFNTLMASQSGVVSYGFIDLDGFSEINNEHGHAFGDKVLDRIVKFGQDFAGNDWEFSREYGQGDEFLLILPNEDKQSGVEVMEEFLNQLHALQPEGESVSASIGVATLPDDGTAEDEVIEKADLAMLNVEQWGGDGVIAYGEEKPTKVIEVWFEEAMLIEQDDLITIQKWMDDGRSIRALKIHNESKGIPNESKSSNTMMSSTKYEGEIKGIVKEIKEWDSSRVTFEMRGHKEQLEEAGF

**Codon optimized gene template for GGDEF_E3 (WP_363463662.1) from *Halogeometricum borinquense* E3**

GTACCCGGGGATCCTCTAGATTTATCT**A**GGAATATCGAACTTGGCTAAAATCTTAAGTAACCCGGATGGCCTTTTTAACACCTTGATGGCATCGCAAAGCGGTGTTGTTAGCTATGGCTTTATTGATCTTGACGGATTTTCGGAAATTAACAACGAACACGGCCACGCATTTGGAGATAAAGTGTTAGATCGTATTGTTAAGTTCGGTCAGGACTTTGCGGGCAACGACTGGGAATTTAGCCGTGAATACGGCCAGGGCGACGAGTTCCTGCTGATTCTGCCGAATGAAGATAAGCAGTCAGGTGTCGAAGTTATGGAGGAATTTCTTAACCAACTTCACGCGCTGCAGCCTGAGGGAGAGAGCGTCAGCGCGTCAATTGGTGTTGCCACTTTACCCGATGATGGTACGGCGGAAGATGAGGTAATTGAAAAAGCGGATCTGGCTATGTTAAATGTAGAACAGTGGGGTGGAGACGGCGTGATTGCCTATGGTGAGGAAAAACCTACCAAGGTGATTGAGGTATGGTTTGAAGAGGCCATGCTGATCGAACAGGACGACCTGATCACGATTCAGAAATGGATGGATGATGGCCGCTCGATCCGTGCCTTAAAGATTCATAATGAATCTAAAGGCATCCCGAACGAATCTAAATCTAGTAATACCATGATGTCGTCGACGAAATATGAAGGCGAGATCAAAGGAATTGTTAAAGAAATCAAAGAGTGGGATTCCTCACGTGTGACTTTTGAAATGCGTGGTCATAAAGAGCAGCTGGAAGAGGCGGGTTTC*CATCACCATCACCATCAC*TGAGCATGCAAGCTTGGCTGTTT

>**MBI5036943.1** **MAG: GGDEF domain-containing protein [Candidatus Micrarchaeota archaeon]**

**MSFWER**MLNKHMENAALLRSGVFAEAQKTRGKLAGFIRKKKRKERAKLPLMLARIGVREPLERGRREMEL

LAAERKGLLADYFKKSTAAKEGSKLILRRLYNPLTGLHSKEFYKEVESELSKPEHANSEIAIASCDLKGF

KNVNDQHGHEAGDKMLKRVAELLEEARRKEGLEHVRIFHPSGDEFIVMAVRTMPEETGAELAVRNLLEQF

QLIKERENERDKKRGAEALHVDETELIGGVSSNVWKKNASVRGITASADTMSYVAKLLGHWGYVSEKQFN

SLPTATQKKILQTLPKVFRREGDRVPREGKAKKGYTRLLCTFSCGRSRVPCHLQNSFYSPIFAKADPRWG

KEKQFLSTTRIDASPKLDLPKGRRKKLNEKQLSPIDYCNNRAKQEYIPLHLLRFAHKSLLAYKTQNLLIK

SEVC

**Template for** [**MBI5036943.1**](https://www.ncbi.nlm.nih.gov/protein/MBI5036943.1)

TAGCGCAAGAGCTTAAATACTTCTGAAACCAAATCAGATTATGTCGTTTTGGGAAAGAATGTTAAACAAGCACATGGAAAACGCGGCATTGCTACGCAGCGGAGTGTTTGCTGAAGCACAGAAAACCAGGGGCAAGCTAGCAGGCTTTATAAGAAAAAAGAAGAGGAAAGAAAGAGCGAAGCTTCCTCTGATGCTAGCGAGAATTGGCGTGCGGGAGCCCCTGGAGAGGGGGCGGCGGGAAATGGAACTACTGGCGGCTGAAAGGAAAGGGCTGCTGGCAGATTATTTCAAGAAAAGTACGGCTGCTAAGGAAGGAAGCAAACTTATTCTGAGGAGGCTCTACAACCCTCTCACAGGCTTGCACAGCAAGGAATTCTATAAGGAGGTGGAGAGCGAGCTTAGTAAACCAGAGCATGCAAACTCTGAAATCGCAATCGCTTCATGTGATTTGAAGGGTTTCAAGAACGTAAACGACCAACACGGACATGAAGCCGGCGACAAGATGTTGAAGAGAGTCGCGGAGTTGCTTGAGGAAGCCCGGAGAAAGGAAGGACTTGAGCATGTCAGGATATTTCATCCCTCAGGGGACGAGTTTATCGTGATGGCGGTCAGAACCATGCCTGAGGAGACGGGAGCAGAGCTGGCGGTGAGGAACCTGCTTGAACAGTTCCAGCTCATAAAGGAAAGGGAGAACGAGAGGGACAAAAAAAGGGGCGCAGAAGCGCTTCACGTGGATGAAACCGAGCTCATCGGCGGGGTTTCCTCGAACGTCTGGAAGAAAAACGCTTCGGTAAGAGGGATAACGGCTTCAGCTGACACGATGAGTTATGTAGCTAAATTGCTGGGCCACTGGGGGTACGTTTCGGAAAAACAGTTCAACTCCCTCCCTACTGCAACCCAGAAAAAGATTCTGCAAACGCTACCCAAAGTATTCCGACGAGAGGGGGATAGGGTACCGAGGGAGGGAAAGGCGAAAAAGGGTTATACGCGGTTGCTTTGTACATTTTCCTGTGGCCGAAGCCGCGTCCCCTGTCATCTCCAAAACTCATTCTATTCACCTATTTTTGCTAAGGCTGACCCGCGCTGGGGGAAAGAAAAACAATTCCTGTCCACAACCCGAATCGACGCCTCACCTAAACTCGATCTTCCGAAAGGCCGTCGCAAAAAACTAAACGAAAAGCAGCTTTCTCCAATCGACTACTGTAACAATAGGGCCAAGCAAGAATATATACCTTTGCATCTCTTGCGCTTTGCTCATAAAAGCTTGCTTGCTTACAAAACCCAAAACTTGCTCATAAAATCCGAAGTTTGCTAAAAGCCTGTTTGTCCTAACCCTTTGG

>**MBI4145595.1** **MAG: GGDEF domain-containing protein [Candidatus Woesearchaeota archaeon]**

MKEEDARRIIQLLASLTSDEQLQLHSLEAELGTIEPHRQLVRGLIFALRQNDTATVRQVIVRIMADIFPS

FMVTLRPLDQEFESVRQQILQQSKIQFEDTEALQQELEQKGMAAAFAERLALAAKRKQITDISAFITARL

GDWRARQSDKKRLLEQEQATLRSIEQTPQPGPNQAVAWADDLDAVERALELVLRKQKDALDALNLALAKQ

RTVERIILDLQQANKMTADALLKVIAGFSKPSEFQELQRIILRHKVALGPEAERVLNLAGTFAVHRTEHV

ETEAAAGGIDFLTQVPNRRTFLQLAEPQLSLARRNRWPAALLVVDIDHFKGINDTYGHDAGDRALALVAR

IMHENVRTSDIVGRWGGEEFVVFLPNTDKIHAAGVAEHLRTLIADKTVTLMSELRKGEPGRANLTVSIGV

AAFPDNADGFNQLFSSADTALYQAKNSGRNRVVSSATA

**Template for MBI4145595.1**

AGACTACAAGCACCACCTGAAGCTTTAAATATAGTTCTTGTCGTATCCCGCCTGTGAAAGAGGAGGACGCGCGACGGATCATCCAGCTTCTCGCCAGCCTCACTTCTGACGAACAGCTGCAATTGCACAGCCTCGAGGCAGAGCTAGGCACGATTGAGCCGCACCGGCAGCTTGTCAGGGGGTTGATTTTCGCGTTGCGCCAGAACGACACCGCGACCGTCCGGCAGGTCATCGTTCGCATCATGGCTGATATCTTCCCTTCGTTCATGGTGACGTTGCGACCGCTCGATCAGGAGTTCGAGTCAGTGCGCCAGCAGATCCTGCAGCAGTCGAAGATCCAGTTCGAGGATACGGAAGCTCTGCAGCAGGAACTCGAACAGAAGGGCATGGCAGCCGCGTTCGCTGAAAGGCTGGCGCTCGCGGCCAAGCGCAAACAGATCACGGACATCTCCGCGTTCATCACCGCGAGATTGGGCGATTGGCGGGCCAGGCAGTCGGACAAGAAACGCCTCCTGGAGCAGGAACAGGCAACGCTTCGGAGCATCGAGCAGACGCCGCAGCCGGGCCCGAACCAGGCCGTCGCGTGGGCCGACGACCTCGATGCCGTCGAACGCGCACTTGAGCTCGTGTTGCGGAAGCAGAAGGACGCTCTCGACGCGTTGAACCTCGCGCTGGCCAAACAGCGTACGGTCGAGCGGATCATCCTCGATCTTCAGCAGGCCAACAAGATGACCGCTGACGCGTTGCTGAAGGTGATCGCGGGGTTCTCCAAGCCTTCAGAATTCCAGGAGCTCCAACGCATAATCCTCAGGCACAAGGTTGCGCTCGGGCCGGAGGCAGAACGCGTGTTGAACCTCGCCGGGACGTTCGCCGTGCACCGGACAGAGCACGTCGAGACCGAGGCTGCCGCGGGAGGGATCGACTTCCTCACGCAAGTCCCGAACAGGCGCACGTTCCTGCAATTGGCAGAACCCCAACTGTCGCTGGCGCGGCGCAACCGTTGGCCCGCCGCGTTGCTGGTGGTCGACATCGACCATTTCAAGGGCATCAACGACACCTACGGCCATGACGCGGGCGACCGCGCGTTGGCGTTGGTCGCGAGGATCATGCACGAGAACGTACGCACCTCGGATATAGTCGGACGCTGGGGCGGCGAGGAGTTCGTCGTGTTCCTGCCGAACACCGACAAGATTCATGCTGCGGGTGTCGCTGAGCATCTGAGGACGTTGATTGCGGACAAGACCGTGACTTTGATGAGCGAACTGCGGAAAGGCGAGCCAGGCCGCGCCAATCTCACCGTCAGCATAGGCGTGGCAGCGTTCCCCGACAACGCGGATGGGTTCAACCAGCTTTTCTCCTCGGCTGACACAGCGCTGTACCAGGCGAAAAACTCGGGCAGGAACCGCGTCGTTTCTAGCGCAACCGCC *CATCACCATCACCATCAC* TGA

**>****MBN1216112.1 MAG: EAL domain-containing protein [Candidatus Lokiarchaeota archaeon]**

MENTSLDKINILIIEDNPGDVKIIEKILSEIETTIYNLVCKVNLSDGIKYALIKKPDIILLDLMLSDSHG

LETLKIIREKVSRIPIVVLTGIDDEEIGVQAVKFGAQDYLVKNYIQSRIFSRVILYAIERSKAEEEIKFL

AYYDYLTKLPNRRYFLERFNATIASAGRYNRISAILFLDIDDFKLINDSLGHKIGDLLLIDISKRLKKCI

RKSNFNYSNKKIETFIDVVARLGGDEFVISLIEISNPEDSSKIAKRIVHELSHPYYVEDHEIYITASIGI

ALYPSDGENTETLLKHADIAMYQAKKSGKNNFKYFTNSMNKSIIKLVSLEKDLRKALDNEEFFLCYQPKM

DLNTGNIIGFEALIRWQQQGKKILLQEDFMSIAEEINLIVPIGYWVIRNIALFLSELKKENLEIPISINI

SLKQLKEVELIKIINNLLIEYNISKELIEFEISESIFLGNLEEIKDNINEINKLGIKIALDNFGEGCSSF

EILKKLKASTIKIGRYFTENILKSNEDASIVSTLISLGHNLNLSVICEGIENFSQLNYLKSKNCDIIQGF

LLSRPVPAEKTRLIMIKEKEGNGIGKKLLRTRRNKNYEQ

**Template for MBN1216112.1**

GCGCTCTAGAAACAGTTTTGGGAAGAATATATGGAGAACACTTCTTTGGATAAAATCAACATCCTGATTATCGAAGATAACCCGGGGGATGTTAAGATTATCGAAAAGATTCTGTCAGAAATCGAGACAACGATCTACAATTTGGTATGTAAGGTGAACTTGAGCGACGGTATTAAATATGCCTTGATTAAGAAACCGGATATCATTCTTTTAGATCTTATGTTAAGTGATAGCCACGGTTTAGAGACGCTTAAGATTATTCGCGAGAAAGTTAGCCGCATCCCAATTGTTGTTCTGACAGGTATTGATGACGAAGAAATTGGAGTGCAGGCTGTTAAGTTCGGGGCACAGGATTATCTGGTAAAAAATTACATCCAGTCGCGCATTTTTAGCCGTGTAATCCTTTATGCAATTGAACGCTCCAAGGCCGAAGAGGAAATTAAATTTCTGGCTTACTATGATTACTTAACGAAGTTACCGAACCGCCGCTACTTTCTGGAACGTTTCAATGCGACCATTGCAAGCGCGGGACGTTATAACCGCATCTCCGCGATCCTGTTCCTTGACATTGACGACTTCAAATTAATCAACGATAGCTTGGGGCATAAAATTGGGGACCTTTTGCTTATCGACATCTCAAAGCGCCTGAAAAAATGCATTCGCAAAAGCAACTTCAACTATTCTAACAAAAAGATTGAGACTTTCATCGACGTCGTGGCTCGCCTGGGGGGCGACGAATTCGTGATTTCGTTAATCGAAATCAGCAACCCTGAGGATTCCTCTAAAATTGCAAAGCGCATTGTACATGAATTATCACATCCTTACTATGTCGAGGACCACGAAATCTACATTACCGCGAGCATCGGTATTGCCTTGTATCCGAGCGATGGGGAAAACACGGAAACACTTTTGAAGCACGCCGACATCGCTATGTACCAGGCAAAAAAGTCAGGGAAAAATAACTTTAAATATTTTACAAACAGCATGAATAAATCAATTATTAAGTTGGTGAGCTTGGAAAAGGATCTTCGTAAGGCTTTAGACAATGAAGAGTTTTTCCTTTGTTACCAGCCAAAAATGGACTTAAACACAGGTAACATTATTGGCTTCGAGGCCTTAATTCGTTGGCAGCAACAAGGTAAGAAAATTCTGTTACAGGAGGATTTTATGTCGATCGCAGAGGAAATTAACCTTATTGTGCCAATTGGATACTGGGTGATCCGCAACATCGCCCTTTTTCTGTCGGAACTTAAAAAGGAGAATTTGGAGATTCCAATTTCTATCAACATTAGCTTGAAACAGTTGAAAGAGGTCGAATTAATCAAAATTATCAACAACTTATTGATTGAATACAATATTTCAAAAGAGTTGATTGAATTCGAAATCTCAGAGTCTATCTTCTTGGGTAATTTAGAAGAGATTAAAGACAACATCAATGAAATCAACAAACTTGGGATTAAAATCGCGCTTGACAATTTTGGAGAAGGGTGTTCCAGCTTCGAGATCCTGAAAAAGCTGAAAGCGTCAACTATCAAGATCGGCCGCTATTTTACGGAGAACATCCTTAAGTCAAACGAGGATGCGAGCATTGTGTCAACTCTGATCTCCCTGGGCCATAATCTGAATTTAAGTGTTATCTGCGAAGGAATTGAGAACTTTAGTCAGCTTAACTACCTGAAGAGCAAGAACTGCGATATTATTCAAGGTTTTCTGTTAAGCCGTCCTGTCCCTGCTGAAAAGACTCGTTTGATTATGATCAAAGAAAAGGAGGGGAACGGGATTGGAAAAAAGCTGCTGCGCACGCGTCGTAACAAAAACTATGAACAG*CATCACCATCACCATCAC*TAAGCTTGCCG

>**AQS32838.1 = KY476720.1** hypothetical protein [uncultured archaeon]

MPKKDYLTEKLNKKVSALDPKFREKITELFSTMHESISLLYEAAIHDEKTGLYNNKFFDTMLDIEFEKAK

RGRQRLCLFVIDIDFFKKINDAYGHLMADKLLKKLAEILQRSLRKSDIIARFGGEEFFIIFPETSIEKAK

FLTSRLREIIKSDKTLKKYSLTISGGLTEFKKSDTKKTLMKRADKALYKAKNSGRDRFVVLK

**Codon optimized gene template for AQS32838.1 = KY476720.1**

GGATCCTCTAGACAATAAGGTTAAAAGTCCTTGTTTCTTAC**G**AT**C**AT**C**ATGCCCAAGAAAGATTACTTGACGGAGAAGCTTAACAAGAAGGTGTCGGCGTTAGACCCGAAATTTCGCGAAAAGATCACAGAGTTGTTCAGCACGATGCATGAGTCCATTTCTTTATTATATGAAGCCGCAATCCATGATGAGAAGACAGGACTTTACAATAACAAATTCTTCGATACGATGCTTGACATCGAATTCGAGAAAGCTAAACGCGGTCGTCAACGTCTGTGTTTATTCGTCATCGATATCGACTTCTTTAAGAAGATCAATGATGCATACGGCCACTTGATGGCCGATAAATTACTTAAAAAGCTTGCCGAGATTCTGCAGCGCTCGCTTCGTAAGAGTGACATTATCGCGCGCTTTGGTGGCGAGGAATTCTTCATCATTTTCCCTGAAACGAGCATCGAAAAGGCTAAGTTTCTTACGAGTCGCCTTCGTGAGATTATTAAGAGCGACAAAACATTAAAGAAGTATTCATTAACAATCAGCGGTGGGCTGACTGAGTTCAAAAAGTCGGACACTAAAAAGACATTGATGAAGCGCGCAGACAAGGCTCTTTATAAAGCAAAGAACTCCGGTCGTGACCGCTTTGTGGTCCTTAAA*CATCACCACCACCACCAC*TGAGCATGCAAGCTT

**>MCR4284784.1 MAG: GGDEF domain-containing protein [archaeon]**

MINKGNSVLNLEFKKEVSKVLKKFKKSLYELHDLATRDDKTGLYNHRFFTNVFEIELEKARRGKQNISLA

MLDIDCFKKYNDKYGHVMGDEILYDLAQTLLKTTRKYDVLARFGGEEFLVLLPETPLSKAKKAAERMRKG

LYKNNKLKKYGITISIGVTEYKDKDTMKKMITRTDKALYQSKQDGRNRVTAI

**Codon optimized gene template for MCR4284784.1**

GGATCCTCTAGAGCAGAAA**GG**AT**G**TATAGTCTATGATCAATAAGGGTAACAGCGTCCTGAACTTGGAATTTAAAAAGGAAGTGTCGAAGGTTTTGAAGAAGTTCAAGAAGTCTTTATACGAGTTGCACGACTTGGCTACACGTGACGACAAGACCGGCTTATACAATCATCGTTTCTTCACAAATGTATTTGAGATTGAGCTGGAAAAAGCACGTCGTGGAAAGCAAAATATTTCTTTAGCGATGCTGGATATTGATTGCTTTAAGAAGTATAATGATAAATACGGACACGTCATGGGTGACGAGATTCTTTATGATTTGGCCCAAACATTGTTAAAGACAACCCGCAAGTATGACGTTTTGGCCCGCTTTGGAGGGGAAGAGTTCCTTGTGCTGTTGCCTGAGACTCCGTTATCGAAGGCCAAAAAGGCTGCGGAACGTATGCGCAAGGGGCTGTACAAGAATAACAAACTGAAGAAGTATGGCATCACAATCAGTATCGGAGTTACGGAATATAAAGACAAGGACACAATGAAGAAGATGATCACGCGCACAGACAAAGCACTTTATCAGTCCAAACAAGACGGTCGCAATCGCGTCACGGCGATC*CACCACCATCATCACCAC*TAAGCATGCAAGCTT

>**KHO53915.1** MAG: GGDEF protein [archaeon GW2011_AR18]

MEEGINKKIEEYEYQIKLMSIFQNFLIDITKKLEIKELAENLLKFLNESFKIDKCSVIINRQRYFLGNLKDEKLIESENRIMKEVYKVSIPYIVKNLKNDTILFDLKNDSEESLLVIPIINDNKIITYVNIYDQPENIHKKDVKLINYFLSKINYAIINSLEYTQVKDKSITDSLTGLYNRNYFIEKLRYESRNFKKYLSIIMTDIDYFKNYNDKNGHQAGDYLLKELAVLLKNNFRTNDIIGRYGGEEFIIILPETDNETGLQVCERLRKSVESYNFKFKENQPNNAVTISIGLMTTVSKEIEVEDLIKEADNNLYKSKTNGRNRATNSIIISKNLSVK

**Codon optimized gene template for KHO53915.1**

GGATCCTCTAGATATTAT**AGGACGT**ATGAACCATGGAGGAAGGTATTAACAAGAAGATTGAAGAATATGAGTACCAAATCAAGTTAATGTCCATTTTCCAGAACTTTTTGATTGACATCACAAAGAAATTGGAGATCAAGGAATTGGCCGAAAATTTGTTAAAATTTCTTAATGAGAGCTTTAAGATCGACAAGTGTAGCGTGATTATTAATCGCCAACGTTATTTTCTGGGAAATCTGAAAGACGAAAAACTTATCGAGAGTGAAAATCGCATCATGAAGGAGGTGTATAAGGTGTCGATCCCATACATTGTTAAGAACTTGAAGAATGATACCATTTTATTTGACTTGAAGAATGACAGCGAGGAGAGTTTGCTGGTCATTCCTATTATTAACGACAACAAGATTATCACATACGTGAATATCTACGATCAGCCTGAAAACATCCATAAGAAGGATGTTAAACTGATCAACTACTTTTTATCTAAAATCAATTATGCCATCATCAATTCCTTGGAGTACACACAGGTGAAGGATAAAAGTATTACTGATTCCTTGACCGGTCTGTATAATCGCAACTATTTCATTGAGAAGTTGCGCTATGAGTCGCGTAACTTCAAGAAATACCTGTCCATCATTATGACAGACATTGATTACTTTAAGAATTACAACGACAAGAATGGGCACCAAGCAGGCGATTATCTGCTTAAAGAGCTTGCTGTGCTTCTTAAGAATAATTTTCGTACGAATGATATTATTGGTCGCTATGGTGGCGAAGAGTTTATCATTATCTTACCCGAGACCGATAATGAGACCGGCTTGCAGGTGTGCGAGCGTCTTCGCAAGTCGGTGGAATCGTACAATTTCAAGTTTAAAGAGAACCAACCGAACAATGCTGTCACGATTTCGATCGGCCTTATGACTACCGTGTCGAAAGAGATTGAAGTTGAAGATTTAATTAAAGAGGCGGATAATAACTTGTATAAATCCAAGACCAATGGCCGTAATCGTGCAACAAATAGCATTATCATCTCTAAGAACTTATCTGTTAAG*CATCACCACCACCATCAT*TGAGCATGCAAGCTT

**>KHO53289.1 MAG: Diguanylate cyclase with PAS/PAC sensor [archaeon GW2011-AR13]**

MHNEECLDKIVKNVIDSNHFVSPITIKRWLDTMKNSNDKIYVTNRILTGLLEKNNLYMQGTIPYIQEVINNEIKNLKNEKSELETLATVDSLTGLFNRRRCVENLNRELKRSAREKKPLSVIMCDLDHFKSINDTYGHDAGDSVLKTISGVAKNNFRGTDILCRIGGEEFFIILPNTAKTNAIYVADRLRRNIEKQPIKIDSQDKPIYITTSIGIYQIGENETVETIMKNADLRLYNAKKSGRNKVCSE

**Codon optimized gene template for KHO53289.1**

GGATCCTCTAGACTTTAA**AGGTAGT**ATTTTTTATGCATAACGAGGAATGTCTTGATAAAATCGTCAAAAACGTTATCGACTCAAACCATTTCGTCTCGCCTATTACAATTAAACGTTGGCTGGACACTATGAAAAACTCCAACGATAAGATCTATGTAACTAACCGCATCCTTACGGGGTTACTGGAAAAGAACAACCTTTACATGCAGGGCACCATTCCCTATATCCAGGAGGTAATTAATAACGAAATCAAGAATCTTAAGAACGAGAAATCGGAGCTTGAGACCTTAGCTACAGTCGATAGCCTGACCGGACTTTTCAATCGCCGCCGCTGCGTGGAAAACCTGAATCGTGAATTGAAGCGCAGTGCGCGCGAGAAGAAACCGCTGTCAGTGATCATGTGTGACTTAGACCATTTCAAGTCCATTAACGATACATATGGACACGACGCGGGAGACTCGGTGCTTAAGACAATTAGTGGAGTAGCTAAGAATAACTTTCGTGGAACGGACATTCTTTGCCGTATCGGCGGTGAGGAATTCTTCATTATTCTTCCCAACACGGCTAAGACGAATGCAATTTATGTAGCGGACCGTCTTCGCCGCAATATTGAGAAACAACCTATTAAAATCGATTCCCAGGACAAACCTATTTACATCACCACCTCCATCGGCATTTATCAGATTGGCGAGAACGAGACAGTCGAAACTATCATGAAGAATGCTGATTTACGCCTGTACAATGCCAAGAAATCTGGTCGTAATAAAGTTTGCTCAGAG*CACCATCACCATCACCAC*TAAGCATGCAAGCTT

**>MBT3408960.1 MAG: diguanylate cyclase [Candidatus Woesearchaeota archaeon]**

**VS**MNKKEVSFTSIENIKKEIDKATSKQGISDEINDISKIIDIDTWVKIQDNFATAINLPVFLIEESGTEIAVSNDFPYFCKLINSKEKGRKACINCRKKNYKELKEEDTNIKYFECHAGLFNIMTPIKINNKMVGAINCSSILKSSRNIAKVIRVAEELNIESVELIDAVNELPIKQREDLVFNGTLLYVLSQTIPGLVNEHKTNIEKINELEIINKFSNEIQSTLQLNETLKIILNKIVKLTNAIESSITIYKEDKDNKILERYTNSKNDLQLFQNIEKQIINKITNEKNLKKIYEFEKIEDEFNIKTTFIKKIKTIPLLMRQKIIGFIFIYLDNIEKDKNLELLQIISSQSAFGIINAQQFKEINTLAIMDKLTNLYNRRYIMEKLDDELKRAQRTERFLSIILMDIDHFSNYNNNNGHPEGDKLLTKISALIKENTREIDIIGRYGGEEIIVLLPDANINEALPVAERIRKAIENYPFANREKQPLGKVTTCIGLVSHRGKNKTKDDLIKLSDDLLYKSKKAGRNKTSYRIIDEKSSEILQSQTHQ

**Codon optimized template for MBT3408960.1**

GGATCCTCTAGATCAAAT**AGGAAGT**GGTGTCAATGAATAAGAAGGAGGTCTCCTTCACAAGTATTGAGAATATTAAGAAGGAAATTGATAAGGCCACCAGTAAGCAAGGCATTAGTGATGAGATTAACGACATTTCCAAGATTATTGATATTGACACATGGGTTAAAATTCAGGATAACTTTGCCACCGCAATCAATCTTCCGGTATTCCTTATTGAAGAGTCCGGTACAGAGATTGCGGTAAGTAATGACTTTCCCTATTTCTGCAAGTTGATCAACTCAAAAGAAAAAGGACGCAAAGCGTGTATTAACTGTCGTAAGAAGAACTACAAAGAGCTGAAAGAAGAAGATACCAACATCAAATATTTCGAATGCCATGCCGGGCTTTTCAACATTATGACCCCGATTAAAATCAACAATAAGATGGTTGGTGCCATTAATTGTAGCAGTATTCTTAAATCGAGCCGCAACATTGCTAAAGTTATTCGCGTAGCAGAAGAACTGAACATTGAAAGTGTCGAGCTGATTGACGCCGTAAACGAATTACCCATTAAACAGCGCGAAGACCTTGTGTTTAATGGCACCTTACTTTACGTCTTATCCCAAACAATCCCAGGTCTGGTGAATGAGCATAAGACGAATATCGAGAAAATTAACGAACTTGAAATTATTAACAAGTTCTCTAATGAGATTCAGTCCACTCTGCAATTGAACGAGACCTTAAAGATTATCTTAAACAAGATTGTGAAACTTACAAACGCCATTGAGAGTAGTATTACTATCTATAAAGAAGACAAAGACAATAAGATTCTGGAACGTTATACAAATTCCAAAAATGACCTTCAATTGTTTCAAAACATCGAAAAACAGATTATTAATAAGATTACAAATGAAAAGAACCTTAAAAAGATCTATGAGTTTGAGAAAATCGAAGACGAGTTCAATATTAAGACCACCTTCATCAAAAAGATTAAGACCATTCCACTTCTGATGCGTCAAAAGATCATCGGCTTCATTTTTATCTATCTGGACAACATCGAAAAAGATAAAAACTTAGAGTTATTACAAATCATTTCCTCCCAGAGTGCTTTTGGCATTATCAACGCACAGCAATTCAAGGAAATCAATACCCTTGCGATTATGGATAAGTTGACCAATCTTTACAACCGCCGTTACATCATGGAGAAACTGGACGATGAGCTGAAGCGCGCTCAGCGCACTGAGCGTTTCTTATCCATTATCCTTATGGACATTGATCATTTTAGCAACTATAACAACAACAACGGTCATCCTGAAGGTGATAAGTTACTGACAAAAATCTCGGCGCTTATTAAAGAGAATACCCGCGAGATCGATATCATTGGACGCTACGGCGGTGAAGAGATTATTGTCTTGCTTCCAGATGCTAATATCAACGAGGCGCTGCCGGTTGCTGAACGCATCCGCAAGGCAATCGAAAATTATCCTTTCGCTAACCGCGAAAAACAACCCCTGGGAAAAGTTACGACCTGTATCGGACTGGTTTCGCATCGTGGAAAGAACAAGACAAAAGACGACTTAATTAAACTTAGCGACGACTTGTTGTATAAATCAAAGAAAGCTGGCCGCAATAAAACAAGCTATCGCATCATTGACGAAAAATCGAGTGAAATCCTGCAGAGCCAGACACACCAG*CATCATCACCATCATCAT*TAAGCATGCAAGCTT

**>HJJ27798.1 MAG TPA: GGDEF domain-containing protein [Methanocorpusculum sp.]**

MATLAQWFYGGKDKEFFIRHSEEIRLANTKTAGTFGIVITALVLLFTIFVCASRLETDLTALFIFTAVLLVLNIYYWVWGREHPKLTVYYIQVILLILIAFLILRDIMEPEEYAVFIPLFLIVMPLLFINPMHKTLISMLVVLAGFACITILVKGPTVYGIYDIVDVCITASFGFFMGQNILRLRIGTIEAYEKLKQSSESEVSKALDMANTDALTGVRSRSAYEKICATLDEKIQEGTASEFALVLCDVNGLKKTNDTMGHEMGDKLIKSCCHEICVVYAHSPVFRIGGDEFVVLLHDSDYMDRKALFEKLHEANMKTNVSFAYGMAEFDSQRDARTQHVFARADAQMYLCKKKMKAARSD

**Codon optimized template for** [**HJJ27798.1**](https://www.ncbi.nlm.nih.gov/protein/HJJ27798.1)

GTACCCGGGGATCCTCTAGATATACATA**GG**CAATAATAAAATGGCCACCCTGGCGCAGTGGTTTTACGGGGGAAAAGATAAGGAATTCTTTATTCGTCACTCCGAAGAAATTCGCCTGGCCAATACCAAGACCGCGGGCACGTTCGGCATTGTAATTACCGCGCTGGTGCTGCTGTTTACGATTTTTGTGTGTGCCTCGCGCCTGGAAACTGATCTGACCGCCTTATTCATTTTTACCGCGGTCCTGCTGGTGCTGAATATCTACTATTGGGTATGGGGTCGCGAACATCCGAAACTTACTGTTTACTACATCCAAGTGATTTTGCTGATTCTTATTGCGTTTTTAATTCTTCGCGATATTATGGAACCCGAGGAATATGCAGTTTTTATCCCTCTTTTCCTGATCGTAATGCCTTTGCTGTTTATTAACCCAATGCATAAAACCTTGATTAGCATGCTGGTGGTGCTGGCAGGATTTGCGTGCATCACGATCCTTGTAAAGGGACCAACGGTGTATGGTATTTATGATATTGTGGATGTGTGCATCACAGCATCGTTTGGTTTTTTTATGGGCCAAAACATTTTGCGTTTACGTATTGGTACGATTGAAGCTTATGAAAAGCTGAAACAGAGTAGCGAAAGCGAGGTAAGTAAAGCCCTGGATATGGCGAACACCGACGCGCTTACCGGCGTTCGCTCGCGTAGTGCGTACGAGAAAATCTGTGCGACGCTGGATGAGAAAATTCAAGAAGGAACCGCCTCTGAATTCGCGTTAGTTCTGTGCGATGTCAATGGTTTAAAGAAAACCAACGATACTATGGGTCACGAGATGGGAGATAAACTTATCAAGAGTTGCTGCCACGAAATTTGTGTGGTGTATGCTCATTCTCCGGTATTCCGTATCGGTGGCGATGAATTTGTTGTACTGCTGCACGATTCGGACTATATGGATCGCAAAGCCCTGTTTGAAAAACTGCACGAAGCTAATATGAAAACCAATGTTTCTTTTGCCTATGGCATGGCCGAATTTGATAGCCAACGCGATGCGCGTACCCAGCATGTCTTTGCCCGTGCGGACGCCCAGATGTACCTTTGCAAAAAGAAAATGAAAGCAGCACGCTCTGAT*CATCACCATCATCACCAT*TAAGCATGCAAGCTTGGCTGTTTTG

**>RJQ22058.1 MAG: GGDEF domain-containing protein [Candidatus Woesearchaeota archaeon]**

MIEQETSAVNRLERIVESAPQVIQVVYALPPSRDAPYTQHCARDVPKSFLGRGPDGVFIDDRIAPNAALYLPRAEGDVFGYTTSMSDAGGRRRLLILSDTQIPLDPEQDSLPPEFRRFYERFIDADQGLSPLPVIEGRQPEYWEVMEEAMREKSRQLKQSRRQLQATTRRFRRLAYMDDNTELPNRRAYWGDMARNVEEAIAYGKKMHVLALDAIGFKKINDEYGQVEGDGVIRQLGEILKRVTRAEDKVYRIGGDEFRVVTDADPYMLKARFEKVVPVATMSVTGPGGTGEEVYLQGVYVGIASFDGARSGVLTEEERRAIFVGLEKKADAAMQEVKAARKTR

**Template for RJQ22058.1**

GTACCCGGGGATCCTCTAGAAAAATCAAA**G**AAGTTGGTGTCTGATGATTGAACAGGAAACAAGCGCGGTTAACCGGTTGGAGCGAATTGTTGAGTCAGCGCCGCAAGTGATTCAGGTTGTCTATGCTCTTCCGCCGTCAAGGGACGCTCCGTACACGCAGCATTGCGCGCGGGATGTGCCCAAATCGTTTCTGGGGCGCGGGCCGGATGGTGTTTTTATTGATGACCGCATCGCGCCGAATGCCGCATTGTATTTGCCAAGGGCAGAGGGCGATGTTTTTGGGTATACGACGTCAATGAGTGATGCGGGCGGGCGTCGCCGTCTTTTGATTTTATCGGACACGCAGATTCCTCTTGACCCCGAGCAGGATTCATTGCCGCCGGAGTTCAGGCGGTTCTATGAGCGGTTCATTGATGCGGACCAGGGGTTGTCTCCGCTTCCGGTAATTGAGGGTAGGCAGCCGGAGTATTGGGAAGTGATGGAAGAGGCGATGCGGGAAAAGTCGCGTCAGCTCAAGCAGTCACGGCGTCAGTTGCAGGCGACAACGCGCCGGTTCCGAAGGCTTGCGTATATGGATGATAATACGGAGTTGCCGAACCGCCGCGCGTACTGGGGAGATATGGCAAGGAATGTGGAAGAGGCGATTGCGTACGGGAAAAAAATGCACGTGCTTGCGCTTGACGCGATAGGGTTCAAGAAGATTAATGATGAGTACGGGCAGGTGGAAGGCGATGGTGTTATCAGGCAGCTTGGTGAGATTTTGAAGCGCGTCACGCGTGCCGAGGATAAGGTGTACCGCATTGGCGGGGATGAGTTTCGCGTGGTGACTGATGCTGACCCGTATATGCTCAAGGCGCGGTTTGAGAAGGTGGTGCCGGTTGCGACGATGAGTGTAACGGGCCCGGGCGGAACAGGTGAGGAAGTTTATCTGCAGGGCGTGTATGTTGGCATTGCAAGTTTTGATGGGGCTCGCTCGGGAGTGTTGACGGAAGAGGAGCGTCGGGCAATTTTTGTCGGTCTTGAGAAGAAAGCGGATGCGGCAATGCAGGAAGTGAAGGCCGCGCGCAAAACACGG*CATCACCATCACCATCAC*TAAGCATGCAAGCTTGGCTGTTTTG

**>UCD02967.1 MAG: GGDEF domain-containing protein [Candidatus Aenigmatarchaeota archaeon]**

**MDSNSIES**MGKSELREYARELQEEIERLQGEVSTDDLTGVLTRKYFIRELGDLIYRSHREDKMASAGVIDIDYFKQYNRDGHELGDRVLKGVAEAGRRVLRQYDLIGRYGGDKGDEFLAGILGWEINGKNIIDRWRREVGTIEVPPGFTTVTITGGVADLNDSPEIQLLRGKRGTDAIMSYKEGEFDRAREYVGVLADWLKTDAKADIDPIELYGALKRLDRSARDFMESMGAEGDLVTYLEDPDFRYPLAATILTNYADRALMAGKRKKKNRIWTPMDISSL

**Template for UCD02967.1**

GTACCCGGGGATCCTCTAGAATTAAT**A**GGTAGTGATATTATGGATAGTAACTCTATAGAATCAATGGGAAAAAGCGAACTAAGAGAATACGCCCGTGAATTACAAGAAGAGATTGAAAGACTCCAGGGCGAAGTATCCACCGATGATCTTACAGGCGTATTGACAAGAAAATATTTTATCAGAGAACTTGGGGATCTAATTTACAGATCTCACCGTGAGGATAAAATGGCCTCAGCTGGTGTTATCGATATTGACTATTTCAAGCAATATAACAGGGACGGACACGAACTCGGTGACAGAGTATTGAAAGGGGTGGCTGAAGCGGGCCGAAGAGTCTTAAGGCAATACGACCTTATCGGAAGGTATGGTGGAGACAAAGGAGACGAGTTCCTTGCCGGAATTCTCGGATGGGAGATAAATGGAAAGAACATAATAGATAGATGGAGACGTGAGGTCGGGACAATTGAGGTGCCGCCGGGGTTCACAACAGTCACAATCACTGGCGGTGTTGCTGATCTAAACGACAGCCCTGAGATCCAACTGTTAAGAGGAAAAAGGGGAACGGATGCGATAATGAGCTATAAAGAAGGAGAGTTTGATAGAGCCAGAGAATACGTGGGTGTATTGGCCGACTGGCTCAAAACTGATGCAAAGGCAGATATTGACCCGATTGAATTGTATGGCGCTTTAAAACGGCTTGATAGAAGCGCCCGCGATTTCATGGAGTCCATGGGGGCCGAAGGGGACTTGGTTACATACCTGGAGGATCCTGATTTCAGGTACCCGCTAGCTGCAACCATTCTGACTAATTATGCAGACAGGGCCCTGATGGCCGGAAAGAGAAAGAAGAAGAACAGAATCTGGACGCCTATGGACATATCATCATTG*CATCACCATCACCATCAC*TGAGCATGCAAGCTTGGCTGTTTTG

**>KYK22704.1 gi|1008839333|gb|KYK22704.1| MAG: hypothetical protein AYK24_00320 [Thermoplasmatales archaeon SG8-52-4]**

MALKEYILNNFQYLIENNPDGIVIHSDGIIRYANKSILTIFGASSADEIIGRNIFDFIQPDYHEAAKIRIAKALNNMEPAPLAEYEAIKLDGAKIYIEILSIPFMRGEAKALQVLVKDISERKRQYKALQYSEEKFRALFQTAPDAILLSNKETNIISLNSALEDMFGYTYDELIGKSWMLLVAKRLRNDRMERIEYIREKLKAGEFLKEEVYGTTKFGKDIPVEIKVGRWQINGEPFYTTIIRDISERREFEAKLKQLADIDHLTGIYNRRAGLILTEQLLKTSRRTNQPITLCYLDLDNFKYVNDSHGHVAGDKVLNAVASLIKDNLRESDIFFRLGGDEFIIVWNNTFLEQGHQQWSRILNKFNEWNKENYSTRLGISAGFVCPDLKKDINIDRIIDIADKEMYKSKGNK

**Codon optimized template for KYK22704.1**

gtacccggggatcctctagagctgaca**a**t**g**tagtaccactatggcgttaaaagaatatattctgaataattttcaatatctgattgaaaataacccggatggcattgtgattcactcggacggaattatccgctacgcaaataaatcgattctgaccatttttggtgcgtccagcgcagatgaaattattggtcgcaatatttttgatttcattcagcctgactaccatgaagctgccaagatccgtatcgctaaagcactgaacaatatggaacctgcacctctggccgagtacgaggcaattaagctggacggggctaaaatttatattgaaattctgtcaatcccattcatgcgcggtgaagccaaagccttgcaagtattggtgaaagatattagtgaacgtaaacgccagtacaaagcgttacaatatagcgaggaaaaattccgcgccctgtttcaaaccgctcctgatgcgattttactgagcaataaagagacgaatatcattagtcttaattcagcgctggaagatatgtttggatatacctatgatgagctgatcggaaagagctggatgcttttagtggccaaacgcctgcgcaacgatcgcatggagcgtattgaatacattcgtgaaaaattaaaggcgggtgaatttctgaaggaagaggtttatgggaccaccaaattcggcaaagatattccggttgagattaaagttggccgttggcagattaacggtgagccgttttacactacgattattcgtgacatctcggagcgtcgcgaattcgaagcgaaacttaaacagctggcggatattgaccatctgactggcatctataaccgtcgcgcgggtttgatcttaaccgagcagctgctgaaaaccagccgccgcaccaatcagccgattaccctgtgttatttagatcttgataatttcaaatatgtaaacgacagccatggccatgtagcaggcgataaggttctgaacgccgtggcaagcctgatcaaagataacctgcgtgaaagcgatattttcttccgtctgggcggtgatgagtttattattgtgtggaacaatacgttcctggaacaggggcatcaacagtggtcgcgtatccttaacaagttcaacgagtggaacaaagaaaattatagcacgcgtttaggcatcagcgccggtttcgtttgtcctgatctgaagaaagacatcaatatcgatcgcattatcgatattgccgataaagaaatgtacaaaagtaaaggcaataaa*catcaccatcaccatcac*taagcatgcaagcttggctgttttg
**Proteins used for construction of the alignment in Figure 1C and Supporting Figure**

WSPR_PSEAER, Q9HXT9 (*Pseudomonas aeruginosa*); PleD_CAUCR, Q9A5I5 (Caulobacter crescentus); CD1420_CLODIF, Q18BU4 (*Clostridioides difficile*); YdeH_Ecoli, P31129 (*Escherichia coli* MG1655); DGCB_CAUCRE, A0A0H3CAN8 (*Caulobacter crescentus*); AdrA_SALTY, Q9L401 (*Salmonella typhimurium* ATCC 14028); PA4332_PSEAE, Q9HW69 (*P. aeruginosa*); VCA0965_VIBCHO, Q9KKY5 (*Vibrio cholerae*); ECA3270_PECCAS, Q6D226 (*Pectobacterium atrosepticum*); DGC1_KOMXYL, O87374 (*Komagataeibacter xylinus*); Y1354_MYCTU Rv1354c/P9WM13 S4 (*Mycobacterium tuberculosis*); YciR_SALTY (*S. typhimurium* ATCC14028); MXAN_2643, Q1D911 (*Myxococcus xanthus*); GSU1658_GEOSL, Q74CL4 (*Geobacter sulfurreducens*); ZMO_1055_1055_strainZM4, ZMO1055 (*Zymomonas mobilis* ZM4); SE0528_STEAS, Q8CTF5 (*Staphylococcus epidermidis*); BifA_PSEAER, Q9HW35 (*P. aeruginosa*).

**Proteins used for construction of the phylogenetic tree in Figure 5D**

WSPR_PSEAER, Q9HXT9 (*Pseudomonas aeruginosa*); PleD_CAUCR, Q9A5I5 (Caulobacter crescentus); ARS29551_1_SPH_KC8, ARS29551.1 (*Sphingomonas* sp. KC8); STRGAL_GGDEF, WP_012961431.1 (*Streptococcus gallolyticus* UCN34); STRSUI_GGDEF, WP_079269016.1 (*Streptococcus suis*); DGCB_CAUCRE, A0A0H3CAN8 (*Caulobacter crescentus*); DGC1_KOMXYL, O87374 (*Komagataeibacter xylinus*); Y1354_MYCTU Rv1354c/P9WM13 S4 (*Mycobacterium tuberculosis*); ECA3270_PECCAS, Q6D226 (*Pectobacterium atrosepticum*); PROMIR_GGDEF, B3F0R5 (*Proteus mirabilis*); VCA0965_VIBCHO, Q9KKY5 (*Vibrio cholerae*); STRHEN_GGDEF, WP_018163948.1 (*Streptococcus henryi*); ZMO_1055_strainZM4, ZMO1055 (*Zymomonas mobilis* ZM4); WP_084652956_1_SPHHAL, WP_084652956.1 (*Edaphosphingomonas haloaromaticamans* strain P3); TMJ20004_1_ALPHA, TMJ20004.1 (Alphaproteobacteria bacterium AP_24); ABQ66580_1_SPHWIT, ABQ66580.1 (*Sphingomonas wittichii* RW1); BifA_PSEAER, Q9HW35 (*P. aeruginosa*); YdeH_Ecoli, P31129 (*Escherichia coli* MG1655); SE0528_STEAS, Q8CTF5 (*Staphylococcus epidermidis*); CD1420_CLODIF, Q18BU4 (*Clostridioides difficile*); PA0861_PSEAE, Q9I580 (*P. aeruginosa*); ); PA4332_PSEAE, Q9HW69 (*P. aeruginosa*); STM1703, STM3388, STM2123, STM2672, STM4551, STM1987, STM1283, STM3375, STM3615 (*Salmonella typhimurium* ATCC14028); AdrA_SALTY, Q9L401 (*S. typhimurium*); EAL_Shenryi_DSM19005, EAL_Shenryi_DSM19005_endPart, WP_018165242.1 (*S. henryi* DSM19005); GSU1658_GEOSL, Q74CL4 (*Geobacter sulfurreducens*); MXAN_2643, Q1D911 (*Myxococcus xanthus*); In VCA0965, the sequence ‘RATNQHDY’ was deleted to optimize the alignment.

**TABLE S1.** Strains used in this study

| **Strain name** | **Genotype** | **Reference** |
| --- | --- | --- |
| ***Escherichia coli* strains** | | |
| TOP10 | F^-^ *mcrA* (*mrr-*hsdRMS-*mcrBC*) 80*lacZ* M15 *lacX74* *recA1 ara139* (*ara-leu*)*7697* *galU galK rpsL* (StrR) *endA1 nupG* | Invitrogen |
| DH5α | F^–^ φ80 *lacZ*ΔM15 Δ(*lacZYA–argF*)U169 *recA1 endA1 hsdR17*(rK–, mK+) *phoA supE44* λ– *thi-1 gyrA96 relA1* | Laboratory collection |
| XL-1 Blue MR | Δ(*mcrA*)183 Δ(*mcrCB-hsdSMR-mrr*)173 *endA1 supE44 thi-1 recA1 gyrA96 relA1 lac* [F′ *proAB lacI*^q^ Δ(*lacZ*)M15 Tn10 (*tet*^R^)] | Laboratory collection |
| TOP10 Vc2Rib | TOP10 attTn*7*::[Vc2 riboswitch–*lacZY*] | (Liu, Kim et al., 2018) |

| **Other bacterial strains** | | |
| --- | --- | --- |
| *Salmonella typhimurium* UMR1 | *S. typhimurium* ATCC 14028s rdar_28°C_, Nal^r^ | (Romling, Bian et al., 1998) |
| *S. typhimurium* UMR1 Δ*yhjH* | *S. typhimurium* ATCC 14028s rdar_28°C_, Nal^r^ Δ*yhjH* | (Simm, Lusch et al., 2007) |
| *Bacillus subtilis* 3A1 | Wild type | Laboratory collection |
| **Archaeal strains** | | |
| *Haloterrigena salinisoli* KCTC4307 | Wild type | (Dong, Mao et al., 2024) |
| *Methanocella arvoryzae* MRE50 | Wild type | (Sakai, Conrad et al., 2010) |

**TABLE S2.** Plasmids used in this study

| **Plasmid name** | **Features** | **Reference** |
| --- | --- | --- |
| **Empty plasmids** | | |
| pBAD28 | pACYC184 origin, M13, Amp^r^, Cm^r^, P_BAD_ promoter, L‑arabinose-inducible | (Guzman, Belin et al., 1995) |
| pBAD30 | pACYC184 origin, M13, Am^r^, P_BAD_ promoter, L‑arabinose-inducible | (Guzman et al., 1995) |
| pSRKGm | pBBR1 origin, Gm^r^, P*_lac_* promoter, IPTG-inducible | (Khan, Gaines et al., 2008) |
| **Plasmid constructs containing** | | |
| **Gene products from archaeal isolates** | | |
| pBAD28-WP_339106355.1 | Codon optimized, includes alternative start-codon, native RBS, inserted in XbaI and SphI sites, C‑terminal 6xHisTag, from *H. salinisoli* KLK7 KCTC4307 | This study |
| pBAD28-WP_339106355.1 (D59A/E60A) | Substitution in GQGDEF motif > GQGAAF | This study |
| pBAD28-WP_363463662.1 | Codon optimized, includes alternative start-codon, native RBS, inserted in XbaI and SphI sites, C‑terminal 6xHisTag, from *H. borinquense* E3 | This study |
| pBAD28-WP_363463662.1 (D79A/E80A) | Substitution in GQGDEF motif > GQGAAF | This study |
| pBAD28-WP_012035199.1 | Codon optimized, includes alternative start-codon, native RBS, inserted in XbaI and SphI sites, C‑terminal 6xHisTag, from *M. arvoryzae* MRE50 | This study |
| pBAD28-WP_012135199.1 (Δ647–end) | Deletion of C-terminal HD-GYP domain | This study |
| pBAD28-WP_012035199.1 (D551A/E552A) | Substitution in GGDEF motif > GGAAF | This study |
| pBAD28-WP_012035199.1 (D680A) | Substitution in HD motif > HA | This study |
| pBAD28-WP_012035199.1 (R454L) | Linker mutation | This study |
| pBAD28-WP_012035199.1 (E465V) | Linker mutation | This study |
| pBAD28-WP_012035199.1 (S472V) | Linker mutation | This study |
| pBAD28-WP_012035199.1 (S472V/D551A/E552A) | Linker mutation and substitution in GGDEF motif | This study |
| **Gene products from metagenome-assembled genomes** | | |
| pBAD28-KY476720.1 | Codon optimized, native RBS, inserted in XbaI and SphI sites, C‑terminal 6xHisTag | This study |
| pBAD28-KY476720.1 (E125A) | Substitution in GGEEF motif | This study |
| pBAD28-KHO53289.1 | Codon optimized, native RBS, inserted in XbaI and SphI sites, C‑terminal 6xHisTag | This study |
| pBAD28-KHO53289.1 (E168A) | Substitution in GGEEF motif > GGAEF | This study |
| pBAD28-KHO53915.1 | Codon optimized, native RBS, inserted in XbaI and SphI sites, C‑terminal 6xHisTag | This study |
| pBAD28-KHO53915.1 (E248A) | Substitution in GGEEF motif > GGAEF | This study |
| pBAD28-MBI5036943.1 | Original sequence, includes alternative start-codon, native RBS, inserted in XbaI and SphI sites | This study |
| pBAD28-MBI5036943.1 (D183A) | Substitution in SGDEF motif > GGAEF | This study |
| pBAD28-MBN1216112.1 | Codon optimized, native RBS, inserted in XbaI and SphI sites, C‑terminal 6xHisTag | This study |
| pBAD28-MBN1216112.1 (D235A) | Substitution in GGDEF motif > GGAEF | This study |
| pBAD28-MBN1216112.1 (E536A) | Substitution in EGI motif > AGI | This study |
| pBAD28-MBT3408960.1 | Codon optimized, native RBS, inserted in XbaI and SphI sites, C‑terminal 6xHisTag | This study |
| pBAD28-MBT3408060.1 (E449A) | Substitution in GGEEI motif > GGAEI | This study |
| pBAD28-MCR4284784.1 | Codon optimized, native RBS, inserted in XbaI and SphI sites, C‑terminal 6xHisTag | This study |
| pBAD28-MCR4284784.1 (E116A) | Substitution in GGEEF motif > GGAEF | This study |
| pBAD28- KYK22704.1 | Codon optimized, native RBS, inserted in XbaI and SphI sites, C‑terminal 6xHisTag | This study |
| pBAD28- KYK22704.1 (D341A) | Alanine substitution in the GGDEF motif > GGAEF | This study |
| KYK22704.1 (D330A) | Alanine substitution in the DxxR I-site motif > AxxR | This study |
| KYK22704.1-D341A (D333A) | Alanine substitution in the DxxR I-site motif >DxxA | This study |
| pBAD28- HJJ[27798.1](https://www.ncbi.nlm.nih.gov/protein/HJJ27798.1) | Codon optimized, native RBS, inserted in XbaI and SphI sites, C‑terminal 6xHisTag | This study |
| pBAD28- HJJ[27798.1](https://www.ncbi.nlm.nih.gov/protein/HJJ27798.1) (D291A) | Alanine substitution in the GGDEF motif > GGAEF | This study |
| pBAD28- RJQ22058.1 | Altered RBS, inserted in XbaI and SphI sites, C‑terminal 6xHisTag | This study |
| pBAD28- RJQ22058.1 (D256A) | Alanine substitution in the GGDEF motif > GGAEF | This study |
| pBAD28- UCD02967.1 | Altered RBS, inserted in XbaI and SphI sites, C‑terminal 6xHisTag | This study |
| pBAD28- UCD02967.1 (D107A) | Alanine substitution in the GGDEF motif > GGAEF | This study |
|  |  |  |
| **Control plasmid constructs** | | |
| pBAD30-*yhjH* | Phosphodiesterase from *S. typhimurium*, EAL domain protein control | (Simm, Morr et al., 2004) |
| pBAD30-*yhjH* (E48A) | Substitution in ELL motif > ALL | (Liu et al., 2018) |
| pBAD30-*yhjH* (E136A) | Substitution in catalytic residue motif ELVE > ELVA | (Simm et al., 2004) |
| pBAD30-*adrA* | Diguanylate cyclase from *S. typhimurium* UMR1, GGDEF domain protein control | (Simm et al., 2004) |
| pBAD30-*adrA* (G288A/G289A) | Substitution in GGEEF motif > AADEF | (Simm et al., 2004) |
| pBAD28-*adrA* | Diguanylate cyclase from *S. typhimurium* UMR1, GGDEF domain protein control | (Li, Cimdins et al., 2019) |
| pBAD28-*adrA* (E290A/E291A) | Substitutions in GGEEF motif > GGAAF | This study |
| pBAD28-*dncV* | Dinucleotide cyclase from *E. coli* ECOR31 | (Li et al., 2019) |
| pBAD28-*dncV* (D129A, D131A) | Substitution in catalytic residues | (Li et al., 2019) |
| pBAD28-*cdaS Bsub* | Diadenylate cyclase from *B. subtilis* 3A1 | This study |
| pBAD28-*cdaS Bsub* (D135A) | Substitution in catalytic residue | This study |
| pSRKGm-*adrA* | Diguanylate cyclase from *S*. *typhimurium* UMR1, for phosphodiesterase assay in the *E. coli* TOP10 Tn*7::*Vc2*lacZY* | This study |

**TABLE S3.** Primers used in this study

| **Name** | **Sequence** | **Gene/vector** |
| --- | --- | --- |
| ***Cloning: vector linearization*** | | |
| pBAD homology Fd | GACCTGCAGGCATGCAAGCT | pBAD28 |
| pBAD homology Re | GACTCTAGAGGATCCCCGGG |  |
| pBAD_Lin.FOR | AGAGTCGACCTGCAGGC | pBAD28 |
| pBAD_Lin.REV | AGAGGATCCCCGGGTAC |  |
| F-pRSK | GCTAGCAATTCGAAAGCAAATTCG | pSRKGm |
| R-pRSK-ATG | CATATGCTGTTTCCTGTGTGAAATTG |  |
| ***Cloning: amplification of insert*** | | |
| Arch_WP_Upstream | GTACCCGGGGATCCTCTAGAGATA | WP_012035199.1 upstream part |
| Arch_WP_re-Upstream | GCGTACGTCCGCCGGG |  |
| Arch_WP_re-Downstream | TTTCCCCGGCGGACGTA | WP_012035199.1 downstream part |
| Arch_WP_Downstream | AAACAGCCAAGCTTGCATGCTTAAT |  |
| SFA_XbaI_Fd | GTACCCGGGGATCCTCTAGA | WP_339106355.1 |
| SFA_SphI_Re | AAACAGCCAAGCTTGCATGC |  |
| KY476720_1_Xba_f* | tttttttgggctagcgaattcgagctcggtacccggGGATCCTCTAGACCTTGTTTCAGG | KY476720.1 |
| KY476720_1_Sph_r* | caggctgaaaatcttctctcatccgccaaaacagccAAGCTTGCATGCTCAGTGGTGGT |  |
| KHO53289_1_Xba_f* | ttttttgggctagcgaattcgagctcggtacccggGGATCCTCTAGACTTTAAAGGTAGT | KHO53289.1 |
| KHO53289_1_Sph_r* | caggctgaaaatcttctctcatccgccaaaacagccAAGCTTGCATGCTTAGTGGTGATG |  |
| KHO53915_1_XbaI_f* | ttttttgggctagcgaattcgagctcggtacccggGGATCCTCTAGATATTATAGGACGT | KHO53915.1 |
| KHO53915_1_Sph_r* | caggctgaaaatcttctctcatccgccaaaacagccAAGCTTGCATGCTCAATGATGGTG |  |
| Woese_Fragment.FOR* | acccggggatcctctAGACTACAAGCACCACCTGAAG | MBI4145595.1 |
| Woese_Fragment.REV* | ctgcaggtcgactctTCAGGCGGTTGCGC |  |
| Microarch_Fragment.FOR* | acccggggatcctctTAGCGCAAGAGCTTAAATACTTCTGAA | MBI5036943.1 |
| Microarch_Fragment.Rev* | ctgcaggtcgactcTTTAGCAAACTTCGGATTTTATGAGCAAG |  |
| MBN1216112_Xba_up* | GCGCTCTAGAAACAGTTTTGGGAAG | MBN1216112.1 |
| down_universal_His_TAA_Hind* | GGCAAGCTTAGTGATGGTGATGG |  |
| MBT3408960_1_Xba_f* | cccgtttttttgggctagcgaattcgagctcggtacccggGGATCCTCTAGATCAAATAGGAAGT | MBT3408960.1 |
| MBT3408960_1_Sph_r* | aggctgaaaatcttctctcatccgccaaaacagccAAGCTTGCATGCTTAATGATGATGG |  |
| MCR4284784_1_Xba_f* | tttttttgggctagcgaattcgagctcggtacccggGGATCCTCTAGAGCAGAAAGGATG | MCR4284784.1 |
| MCR4284784_1_Sph_r* | ggctgaaaatcttctctcatccgccaaaacagccAAGCTTGCATGCTTAGTGGTGATGA |  |
| IVC-XbaI-ATG-Forward_universal | GTACCCGGGGATCCTCTAGA | HJJ27798.1 RJQ22058.1 UCD02967.1  KYK22704.1 |
| IVC-SphI-Stop-Reverse_universal | CAAAACAGCCAAGCTTGCATGC |  |
| cdaS_Bsub_IVC_Fd_originalRBS_FLgene | cccggggatcctctagagtcAAATTTAAACAGGAGATGAAGGCT | *cdaS* from *Bacillus subtilis* 3A1 |
| cdaS_Bsub_IVC_Re | tgcatgcctgcaggtcttaatggtgatggtgatggtgCGTTCTTGGTGAAATTAACGGAT |  |
| adrA_pSRKGm_IVC_Fd | cacacaggaaacagcatATGTTCCCAAAAATAATGAATGATGAAAATTTTTACCGA | *adrA* from *S. typhimurium* UMR1 |
| adrA_pSRKGm_IVC_Re | ctttcgaattgctagctcagtgatggtgatggtgatgTGCCGCCACTTCGGTGC |  |
| ***Site-directed mutagenesis*** | | |
| WP_012035199_1_DE551AA_F | GCAAAAcgCcgCACCTCCGGTACGGCAGAC | WP_012035199.1 |
| WP_012035199_1_DE551AA_R | GGAGGTGcgGcgTTTTGCATCCTTCTTCCC |  |
| WP_012035199_1_D680A_F | TTGCACGcgCTTGGGAAGATCGGG | WP_012035199.1 |
| WP_012035199_1_D680A_R | CCCAAGcgCGTGCAAACGAGCAAG |  |
| WP_012035199_1_R454L_F | AACGAGCtgAACACGACGCTTGAAGAGTTGCA | WP_012035199.1 |
| WP_012035199_1_R454L_R | CGTGTTcaGCTCGTTGATATACTTCCCTTGG |  |
| WP_012035199_1_E465V_F | CGCTCGGtgCGCCGTTTGCAGGAAATGTCCT | WP_012035199.1 |
| WP_012035199_1_E465V_R | ACGGCGcaCCGAGCGCTGCAACTCTTCAAG |  |
| WP_012035199_1_S472V_F | GAAATGgtgTACCGCGATGGCTTGACAGGA | WP_012035199.1 |
| WP_012035199_1_S472V_R | GCGGTacaCCATTTCCTGCAAACGGCGTTCC |  |
| HS_KLK7_QGAAF_f | AGGGTGcgGcGTTTTTGATGGTCTTACCTGATG | WP_339106355.1 |
| HS_KLK7_QGAAF_r | AAAACgCcgCACCCTGTCCGTATTCCCGAT |  |
| HB_DE59AA_Fd | AGGGCGcCGcGTTCCTGCTGATTCTGCCGAATGAAG | WP_363463662.1 |
| HB_DE59AA_Re | AGGAACgCGgCGCCCTGGCCGTATTCACGG |  |
| KY476720_D125A_F** | CGCTTTGGTGGCGcGGAATTCTTCATC | KY476720.1 |
| KY476720_D125A_F** | CGCGATAATGTCACTCTTACGAA |  |
| KHO53289_G168A_F** | GTATCGGCGGTGcGGAATTCTTCATT | KHO53289.1 |
| KHO53289_G168A_R** | GGCAAAGAATGTCCGTTCCACGAAA |  |
| KHO53915_D248A_Fd** | AGAGTTTATCATTATCTTACCCGAGACCGATAATGAGA | KHO53915.1 |
| KHO53915_D248A_Re** | gCGCCACCATAGCGACCAATAATATCATT |  |
| MA_GAAF_F** | CTCAGGGGcCGcGTTTATCGTGAT | MBI5036943.1 |
| MA_GGAEF_R** | GGATGAAATATCCTGACATGCTC |  |
| MBN1216112_D235A_F** | CCTGGGGGGCgcgGAATTCGTGATTT | MBN1216112.1 |
| MBN1216112_D235A_Rev** | CGAGCCACGACGTCG |  |
| MBN1216112_E536A_F** | GTTATCTGCGcAGGAATTGAGAAC | MBN1216112.1 |
| MBN1216112_E536A_Rev** | ACTTAAATTCAGATTATGGCCCA |  |
| MBT_3408960_D449A_Forw** | ACGGCGGTGcAGAGATTATTGTCTT | MBT3408960.1 |
| MBT_3408960_D449A_rev** | AGCGTCCAATGATATCGATCTCGC |  |
| MCR4284784_D116A_Forw** | CTTTGGAGGGGcAGAGTTCCTTGTG | MCR84784.1 |
| MCR4284784_D116A_Rev** | CGGGCCAAAACGTCATACTTGCGG |  |
| KYK22704.1-D341A-fw | GGCGGTGcgGAGTTTATTATTGTGTGGAACAATAC | KYK22704.1 (GGDEF-GGAEF) |
| KYK22704.1-D341A-rev | AAACTCcgCACCGCCCAGACGGAAGAAAAT |  |
| KYK22704.1-R330A-fw | AACCTGgcgGAAAGCGATATTTTCTTCCGTCTG | KYK22704.1 (RxxD-AxxD) |
| KYK22704.1-R330A-rev | GCTTTCcgcCAGGTTATCTTTGATCAGGCTTGC |  |
| KYK22704.1-D333A-fw | GAAAGCgcgATTTTCTTCCGTCTGGGCGGTGA | KYK22704.1 (RxxD<RxxA) |
| KYK22704.1-D333A-rev | GAAAATcgcGCTTTCACGCAGGTTATCTTTG |  |
| HJJ[27798_1](https://www.ncbi.nlm.nih.gov/protein/HJJ27798.1)_D291A_fw | GTGGCGcgGAATTTGTTGTACTGCTGCACGATT | HJJ27798.1 |
| HJJ[27798_1](https://www.ncbi.nlm.nih.gov/protein/HJJ27798.1)_D291A_rev | CAAATTCcgCGCCACCGATACGGAATACCG |  |
| RJQ22058_1_D256A_fw | GCGGGGcgGAGTTTCGCGTGGTGACTGATGC | RJQ22058.1 |
| RJQ22058_1_D256A_rv | GAAACTCcgCCCCGCCAATGCGGTACACCT |  |
| UCD02967_1_D107 A_fw | AAGGAGcgGAGTTCCTTGCCGGAATTCTCGG | UCD02967.1 |
| UCD02967_1_D107 A_rv | GGAACTCcgCTCCTTTGTCTCCACCATACCTTC |  |
| adrA-GGAAF-fw | CGGCGcgGcgTTTGCGGTGATTATGTGCGGAAC | AdrA from *S. typhimurium* UMR1 |
| adrA-GGAAF-rev | GCAAAcgCcgCGCCGCCAAAGCGCCCGATAA |  |
| cdaS_Bsub_D135A_IVC_Fd | AGCGCCGgCATGAAGAGGGTTTCCGGGAAA | *cdaS* from *Bacillus subtilis* 3A1 |
| cdaS_Bsub_D135A_IVC_Re | CTTCATGcCGGCGCTTTATTAGTGAGGGAAAACAAACTC |  |
| ***Deletion of C-terminal domain*** | | |
| WP_IVC_del_Fd | gcgattatatggatcacggtCATCACCACCACCATCATTAAGCATGC | WP_012035199.1 |
| WP_IVC_del_Re | ATCGACAATCACCGTCAGCACA |  |
| ***Screening and sequencing*** | | |
| F-pBAD-screen | GTCTATAATCACGGCAGAAAAGTCCAC | pBAD28 |
| R-pBAD-screen | CTGTTTTATCAGACCGCTTCTGC |  |
| WP_012035199_1_InternalSeq | TGAATCAACGTTCTAACGTGACG | WP_012035199.1 |
| F-screen-pRSK | CTATTTAACGACCCTGCCCT | pSRKGm |
| R-screen-pRSK | GCGGATAACAATTTCACACAGG |  |
| ***Primers for qRT-PCR*** | | |
| qL10_KLK7_f | GGTTCGATTCAGGTCATGGAGG | *rplJ* of *H. salinisoli* KLK7 |
| qL10_KLK7_r | GGGAAGCTCGCGTTGATCGC |  |
| qGGDEF_KLK7_f | TTTGGTTTGAAGAAGCCATGTTGG | *GGDEF_KLK7* of *H. salinisoli* KLK7 |
| qGGDEF_KLK7_r | TCTTATCACCTCTCATCTCAAACC |  |
| qL10_MRE50_f | GCGAGAAGGCGAAGGAACTGG | *rplJ* of *M. arvoryzae* MRE50 |
| qL10_MRE50_r | TAGTGTCCTTGATGACTACTTTGC |  |
| qHydro_MRE50_f | GTCTACACCAGAGTCCTGTCC | *vhtA* of *M. arvoryzae* MRE50 |
| qHydro_MRE50_r | GGGGAGATGATCTGGTAGCCG |  |
| qGGDEF_MRE50_f | TTCTGGATCGTCGGCCAGGTG | *GGDEF_MRE50* of *M. arvoryzae* MRE50, GGDEF |
| qGGDEF_MRE50_r | TGCAACTCTTCCAGCGTGGTGT |  |
| qHDGYP_MRE50_f | GAGCGAGACTATATGGACCACG | *GGDEF_MRE50* of *M. arvoryzae* MRE50, HD-GYP |
| qHDGYP_MRE50_r | GTGCGATATGATTCATGTGGAGG |  |

Modified nucleotides are shown in lowercase, and template-complementary sequences are shown in uppercase. Homology regions for in vivo cloning are underlined.
* denotes primers used for conventional cloning;
** denotes primers used for the Q5 Quick Change kit or an equivalent reaction assembled from the individual components
